# 14,15-EET induces the infiltration and tumor-promoting function of neutrophils to trigger the growth of minimal dormant metastases

**DOI:** 10.18632/oncotarget.9709

**Published:** 2016-05-30

**Authors:** Jing Luo, Xin-Xia Feng, Chao Luo, Yu Wang, Dong Li, Yu Shu, Shan-Shan Wang, Jian Qin, Yong-Chao Li, Jiu-Ming Zou, De-An Tian, Gui-Mei Zhang, Zuo-Hua Feng

**Affiliations:** ^1^ Department of Biochemistry and Molecular Biology, Wuhan University, School of Basic Medicine, Hubei, Wuhan 430030, People's Republic of China; ^2^ Tongji Hospital, Tongji Medical College, Huazhong University of Science and Technology, Hongshan, Wuhan 430030, People's Republic of China

**Keywords:** neutrophils, recruitment, metastases, dormancy, epoxyeicosatrienoic acids

## Abstract

Infiltrating neutrophils are known to promote in thedevelopment of tumor. However, it is unclear whether and how neutrophils areinvolved in triggering the growth of dormant metastases. Here we show that14,15-epoxyeicosatrienoic acid (14,15-EET) can trigger the growth of dormantmicrometastases by inducing neutrophilic infiltration and converting neutrophilfunction. 14,15-EET triggered neutrophil infiltration in metastatic lesions byactivating STAT3 and JNK pathways to induce the expression of human IL-8 andmurine CXCL15 in corresponding tumor cells. The continuous expression ofhIL-8/mCXCL15 was maintained by the sustained and enhanced activation of JNKpathway. 14,15-EET up-regulated miR-155 expression by activating STAT3 and JNKpathways. miR-155 in turn down-regulated the expression of SHIP1 and DET1, thusaugmenting the activation of JNK and c-Jun. Moreover, the function ofneutrophils was converted from tumor-suppressing to tumor-promoting by14,15-EET *in vivo*. By inducing the production of G-CSF/IL-6 *in vivo*, 14,15-EET induced the enhancement of STAT3 activation in neutrophilsto increase MMP-9 expression and decrease TRAIL expression. Neutrophil-derivedMMP-9 was required for 14,15-EET to induce angiogenesis during the growth ofdormant micrometastases. Depleting neutrophils or inhibiting hIL-8/mCXCL15up-regulation resulted in the failure of 14,15-EET to promote the developmentof micrometastases. These findings reveal a mechanism through which theinfiltration and tumor-promoting function of neutrophils could be induced totrigger the growth of dormant metastases, which might be a driving force forthe tumor recurrence based on dormant metastases.

## INTRODUCTION

The minimal dormant metastases have been recognized as the main cause of cancer recurrence [1–3]. Tumor dormancy is maintained in the microenvironment unfavorable for tumor cell proliferation [3, 4]. When microenvironmental conditions shift to support tumor expansion, dormant tumors can resume active growth and progression [3]. The failure of angiogenesis is a factor that contributes to the maintenance of the dormant state [1, 5]. Accordingly, the activation of angiogenesis has been implicated as a trigger for the initiation of growth by dormant tumor cells [1, 5]. However, the mechanisms underlying the angiogenesis during the growth of dormant metastases remain poorly understood.

Polymorphonuclear leukocytes (PMNs or neutrophils) can produce matrix metalloproteinase-9 (MMP-9) to initiate angiogenesis in the development of tumor [6]. The potent angiogenic factors such as VEGF and FGF2 are usually sequestered in an inactivated form to the extracellular matrix (ECM) [7]. MMP-9 remodels ECM and releases VEGF and FGF2, which then act on endothelial cells to prompt re-vascularization [7]. Although many types of cells can produce MMP-9 [3], infiltrating neutrophils have been found to trigger angiogenesis in the development of tumor [6, 8], and support metastatic initiation [9, 10]. On the other hand, naive neutrophils have the capacity to suppress angiogenesis by secreting neutrophil elastase (NE) to degrade VEGF and FGF2, and releasing TRAIL to induce vascular disruption [7, 11]. During tumor progression, neutrophil function is converted from tumor-suppressing to tumor-promoting [12, 13], thus producing more MMP-9 but releasing much less NE and TRAIL [12]. However, despite clear evidence that neutrophils promote angiogenesis during tumor development, the relationship between neutrophils and dormant metastases remains unclear. The failure of angiogenesis in dormant metastases may hint at the absence of neutrophils in these metastatic lesions. So far little is known about whether and how neutrophils are recruited to the dormant metastases to initiate angiogenesis.

14,15-epoxyeicosatrienoic acid (14,15-EET) is one of four regioisomeric EETs (5,6-EET, 8,9-EET, 11,12-EET, and 14,15-EET) that correlate with various biological processes, including vasorelaxation, inflammation, and the response to tissue injury [14]. In recent years, however, 14,15-EET has been found to promote tumor cell proliferation, the growth of primary tumors, and tumor metastasis [14–16]. 14,15-EET can also stimulate the endothelial cells to express VEGF and FGF2 [17, 18]. These findings raised the question of whether the dormant metastases might be influenced by the elevation of the circulating 14,15-EET in absence of primary tumor, since EET levels can be directly influenced by nutrients and inflammatory processes [14, 15]. Here, we show that 14,15-EET could induce neutrophilic infiltration in metastatic lesions and the conversion of neutrophil function, thus triggering the growth of minimal dormant metastases. Neutrophil-derived MMP-9 is required for 14,15-EET to induce angiogenesis during the growth of dormant metastases. In neutrophil-depleted mice or in MMP-9^−/−^ mice, 14,15-EET failed to induce angiogenesis in metastatic lesions and metastases development.

## RESULTS

### Growth of minimal dormant metastases can be triggered by 14,15-EET

**Figure 1:**
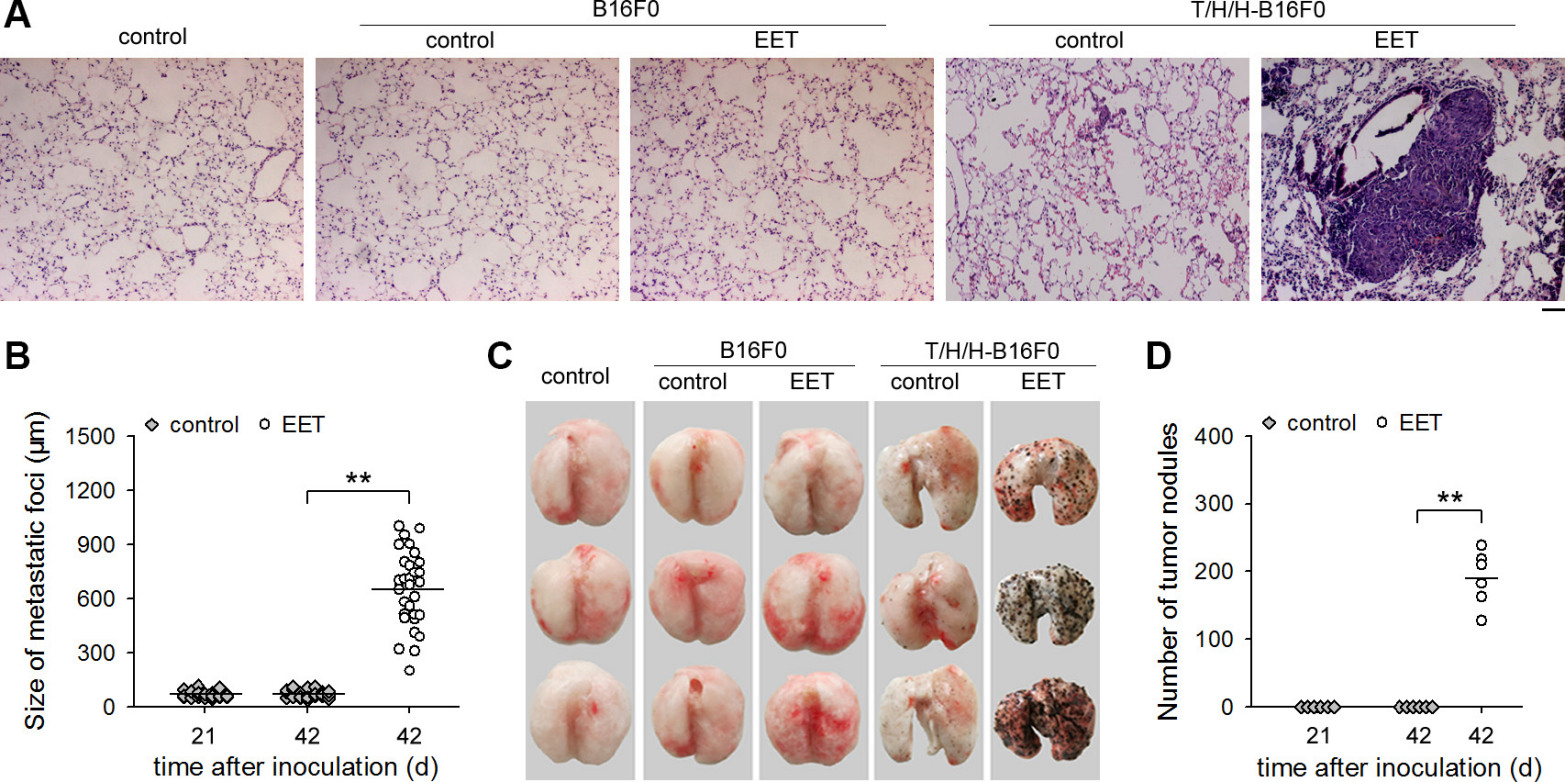
14,15-EET triggers the growth of minimal dormant metastases Mice (*n* = 6 per group) were inoculated with B16F0 cells (A, C) or T/H/H-B16F0 cells (**A**–**D**), and then were untreated or treated with 14,15-EET. The mice were sacrificed at the indicated time points (B, D) or on d42 (A, C) after inoculation. (A) Representative images of the sections of lung tissues after H&E staining (100 × magnification, Bar, 50 μm). (B) Metastatic foci in tissue sections were measured as described in Methods. (C and D) Metastatic nodules on the surface of lungs (C) were counted (D). **p < 0.01.

Non-metastatic tumor cells could acquire the invasive capacity after stimulation by extracellular signal molecules. However, these cells could only form the dormant micrometastases, but not macroscopic metastases, after extravasation [19, 20]. To determine whether 14,15-EET could trigger the growth of dormant micrometastases, we inoculated the mice with non-metastatic tumor cells (B16F0, HepG2, and MCF-7 cells) that were pretreated with TGF-β1/H_2_O_2_/HOCl (T/H/H) to induce the invasive capacity, and treated the mice with 14,15-EET (Supplementary Figure S1). The growth of dormant micrometastases was triggered by 14,15-EET (Figure 1A, 1B, and Supplementary Figure S2A), resulting in the formation of visible metastatic nodules on the surface of lung (Figure. 1C, 1D, and Supplementary Figure S2B). 14,15-EET promoted the growth of metastases in a dose-dependent manner (Supplementary Figure S2C). The other three regioisomers of EET were much less effective (Supplementary Figure S2D). Furthermore, the effect of 14,15-EET was abolished by 14,15-EEZE, an antagonist of 14,15-EET [14], but was not mimicked or influenced by 14,15-DHET, the metabolite of 14,15-EET [14] (Supplementary Figure S2E).

### 14,15-EET-induced neutrophilic infiltration is required for the growth of dormant metastases

**Figure 2:**
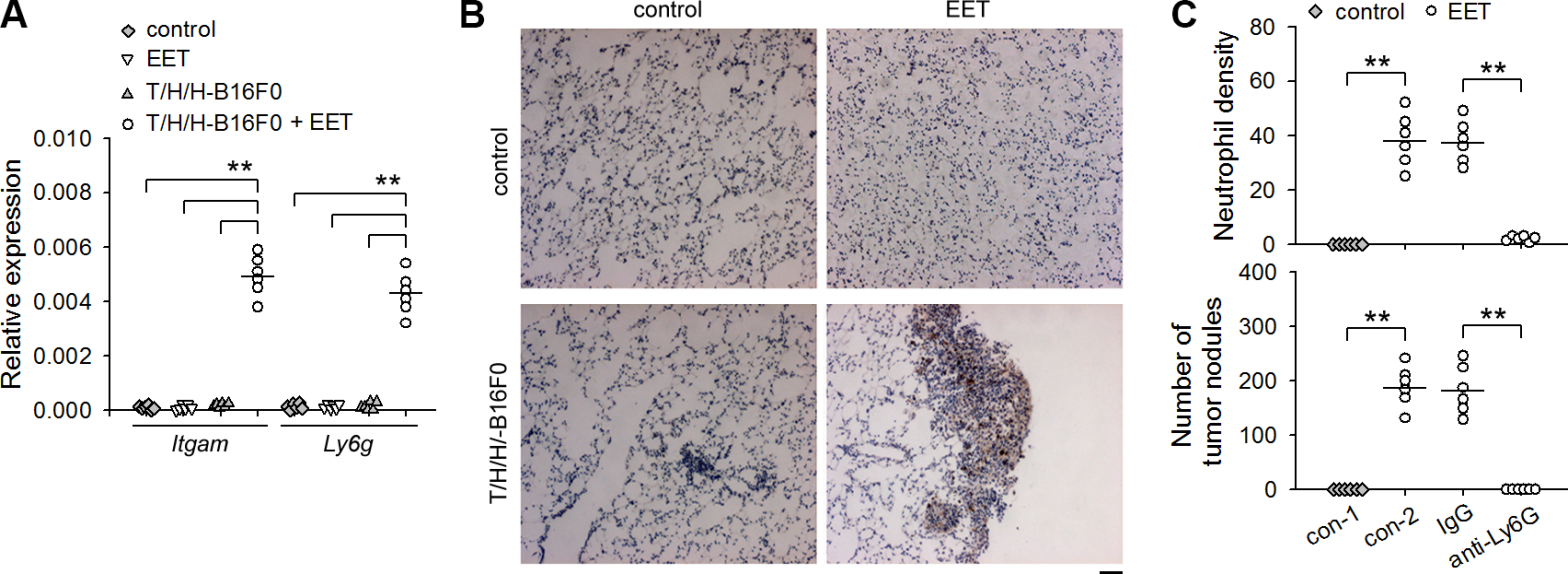
14,15-EET induces neutrophilic infiltration to promote the development of metastatic lesions Control mice (**A** and **B**) and the mice inoculated with T/H/H-B16F0 cells (A to C) were untreated or treated with 14,15-EET. The mice (*n* = 6 per group) were sacrificed on d42 after inoculation. (A) The expression of *Itgam* (CD11b) and *Ly6g* (Ly6G) genes in lung tissues was detected by real-time RT-PCR. (B) The sections of lung tissues were subjected to immunohistochemical staining for identifying the infiltration of neutrophils. Bar, 50 μm. (**C**) Anti-Ly6G antibody was used to deplete neutrophils *in vivo* when the mice were treated with 14,15-EET. Neutrophil density in lung tissue sections was determined after immunohistochemical staining as described in Methods (upper). Metastatic nodules on the surface of lungs were counted (lower). **p < 0.01.

To clarify whether neutrophils might be involved in 14,15-EET-induced growth of dormant metastases, we detected the infiltration of neutrophils in the lung tissues harboring dormant metastases (B16F0 model) by detecting the mRNAs of CD11b and Ly6G (Figure 2A), immunohistochemical staining (Figure 2B), and immunofluorescence (Supplementary Figure S3A). Neutrophils were not observed in the lung tissues harboring dormant metastases. The treatment with 14,15-EET induced the infiltration of neutrophils in the metastatic lesions, but not in control lung tissues. To ascertain the role of neutrophils in 14,15-EET-induced growth of dormant metastases, we depleted neutrophils *in vivo* (Supplementary Figure S3B). Neutrophil depletion abrogated the promoting effect of 14,15-EET on the development of metastatic lesions into visible metastatic nodules in the metastatic models using B16F0 cells (Figure 2C), HepG2 cells and MCF-7 cells (Supplementary Figure S3C). Similarly, depleting neutrophils also suppressed the promoting effect of 14,15-EET on the metastatic growth of B16F1 cells, a metastatic clone of melanoma B16 (Supplementary Figure S3D). These results suggested that 14,15-EET induced neutrophilic infiltration to promote the development of metastatic lesions.

### 14,15-EET promotes hIL-8/mCXCL15 expression in tumor cells to recruit neutrophils

**Figure 3:**
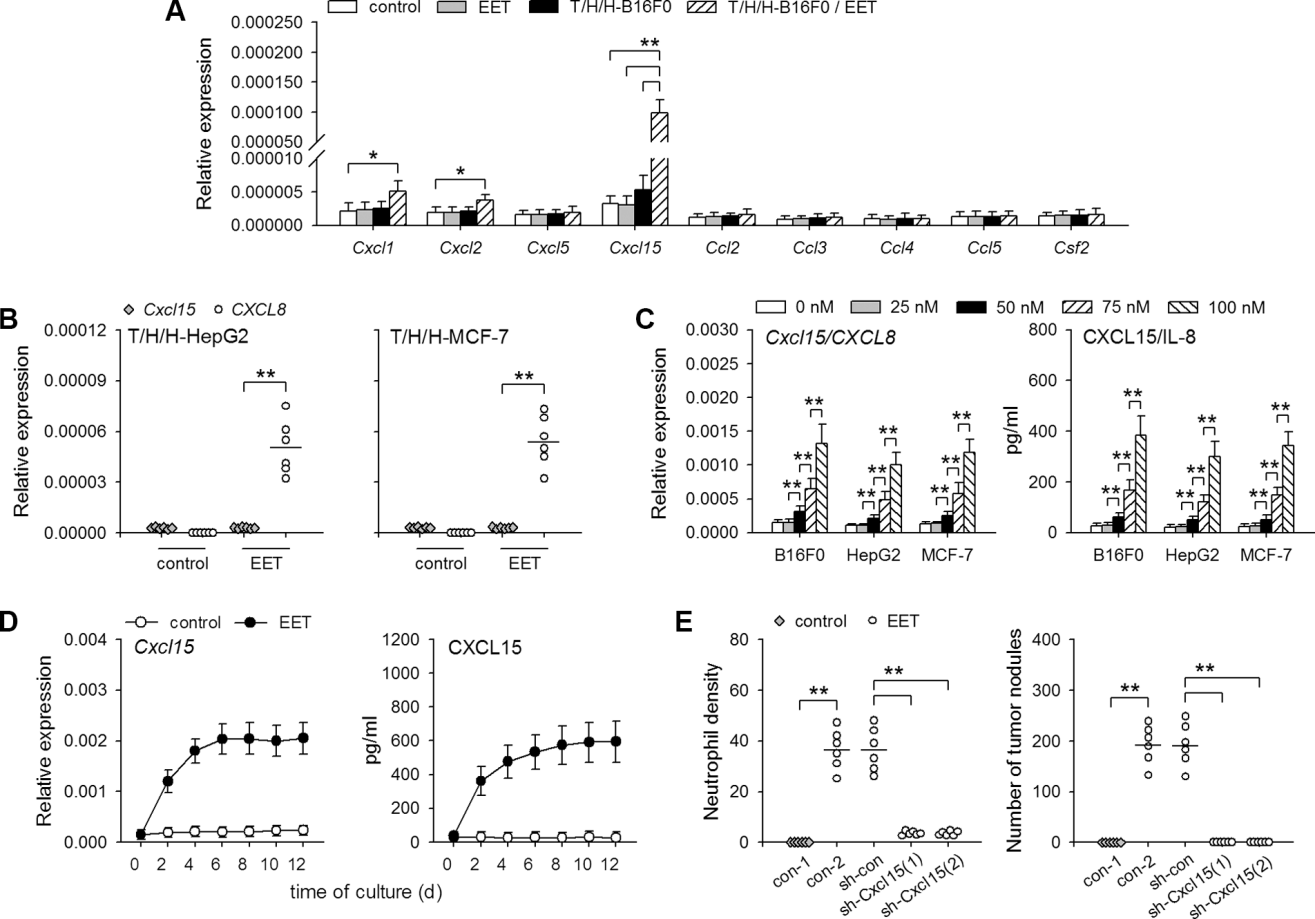
14,15-EET induces hIL-8/mCXCL15 expression in tumor cells (**A**) Control mice and the mice inoculated with T/H/H-B16F0 cells were untreated or treated with 14,15-EET. The mRNA levels of the indicated mouse genes in lung tissues were detected by real-time RT-PCR on d42 after inoculation (*n* = 6 per group). (**B**) Mice inoculated with T/H/H-HepG2 cells or T/H/H-MCF-7 cells were untreated or treated with 14,15-EET. On d42 after inoculation, the mRNAs of mouse *Cxcl15* gene and human *CXCL8* (*IL8*) gene in lung tissues were detected by real-time RT-PCR, and both were quantified against mouse *Gapdh* mRNA (*n* = 6 per group). (**C**) Tumor cells were untreated or treated with 14,15-EET for 48 h at the indicated concentrations. The expression of *Cxcl15*/*CXCL8* gene was detected by real-time RT-PCR and ELISA (*n* = 6 per group). (**D**) B16F0 cells were untreated or treated with 14,15-EET. The expression of *Cxcl15* gene was detected by real-time RT-PCR and ELISA at the indicated time points. (**E**) B16F0 cells, untransfected or transfected with the indicated vectors, were inoculated to mice after T/H/H-treatment. The mice, untreated or treated with 14,15-EET, were sacrificed on d42 after inoculation. Neutrophil density in lung tissue sections was determined after immunohistochemical staining (left). Metastatic nodules on the surface of lungs were counted (right). **p < 0.01.

To analyze the mechanisms underlying the recruitment of neutrophils during the growth of dormant metastases, we investigated whether 14,15-EET might induce the production of neutrophil chemoattractants by detecting the expression of the genes coding for CXCL1, CXCL2, CXCL5, CXCL15, CCL2, CCL3, CCL4, CCL5, GM-CSF [21–23]. The results showed that 14,15-EET substantially increased the expression of *Cxcl15* gene (also known as *Il8*, *lungkine*. http://www.ncbi.nlm.nih.gov/gene/?term=Cxcl15), but not the genes coding for other neutrophil chemoattractants, in the lung tissues harboring metastases from T/H/H-B16F0 cells (Figure 3A). Interestingly, when mice were inoculated with T/H/H-HepG2 or T/H/H-MCF-7 cells, 14,15-EET-treatment increased the levels of human *IL8* mRNA, but not mouse *Cxcl15* mRNA in the lung tissues (Figure 3B), suggesting that 14,15-EET induced hIL-8/mCXCL15 expression in tumor cells, but not non-cancerous cells. Indeed, 14,15-EET induced CXCL15 expression in B16F0 cells, and IL-8 expression in HepG2 and MCF-7 cells, in a dose-dependent manner at physiologically relevant concentrations (1 to 100 nM, ref. 24) (Figure 3C). The prolonged stimulation with 14,15-EET induced the continuous expression of CXCL15 (Figure 3D). CXCL15 expression in B16F0 cells was not substantially increased by other regioisomers of EET (Supplementary Figure S4A). 14,15-EET did not efficiently induce the expression of other neutrophil chemoattractants in B16F0 cells (Supplementary Figure S4B). Same results (inducing IL-8 but not other neutrophil chemoattractants) were obtained in HepG2 and MCF-7 cells (data not shown).

To clarify the crucial role of tumor cell-derived hIL-8/mCXCL15, we used shRNA to inhibit the up-regulation of hIL-8/mCXCL15 (Supplementary Figure S4C). When the up-regulation of CXCL15 (B16F0 cells) and IL-8 (HepG2 cells) was inhibited, 14,15-EET failed to promote the neutrophilic infiltration and the development of micrometastases (Figure 3E and Supplementary Figure S4D), suggesting that 14,15-EET indeed promoted neutrophilic infiltration in metastatic lesions by up-regulating neutrophil chemoattractant expression in tumor cells.

### 14,15-EET promotes hIL-8/mCXCL15 expression in tumor cells by activating STAT3 and JNK pathways

**Figure 4:**
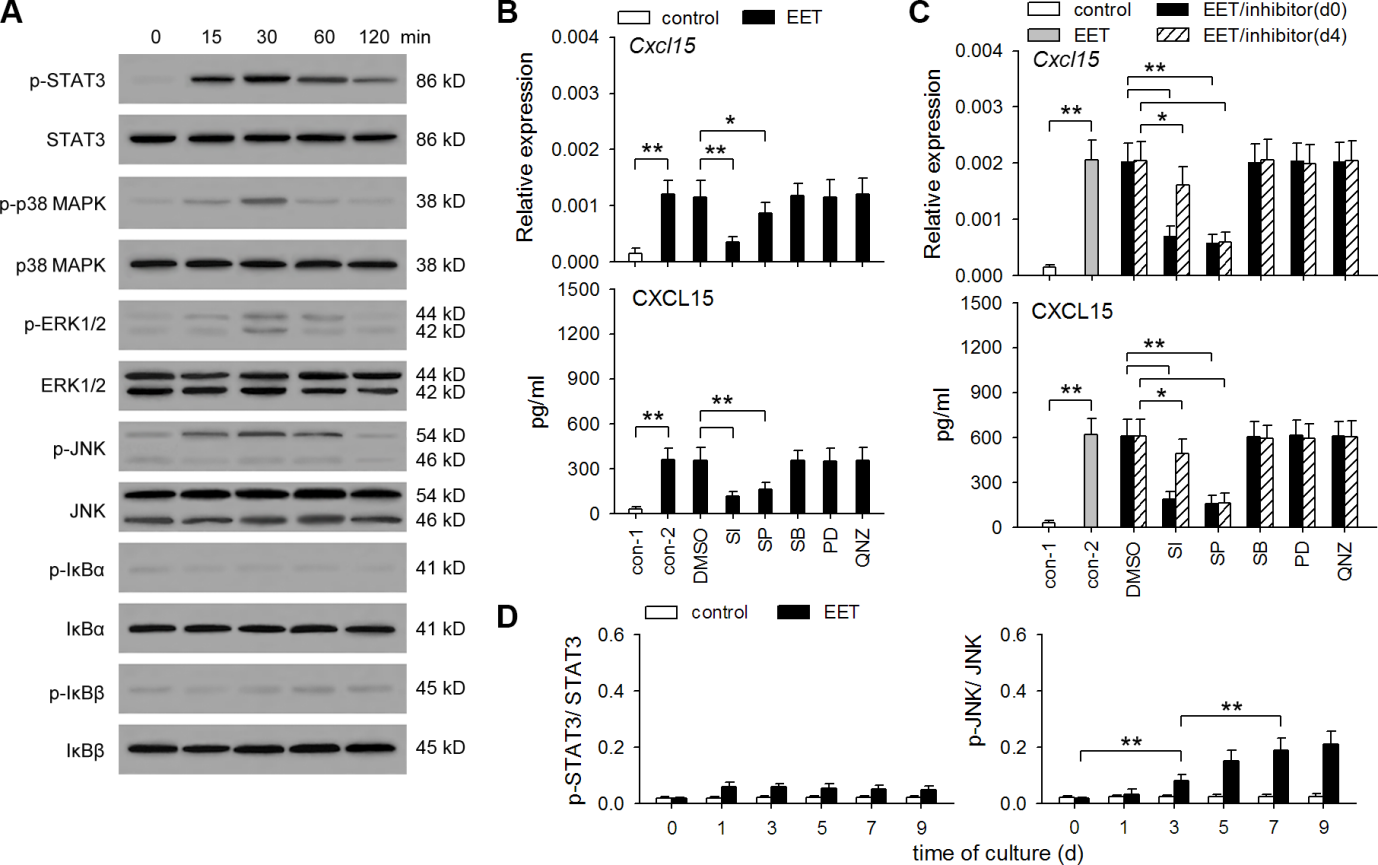
14,15-EET activates STAT3 and JNK pathways to up-regulate CXCL15 expression (**A**) B16F0 cells were stimulated with 14,15-EET (100 nM). The phosphorylation of STAT3, p38 MAPK, ERK, JNK, IκBα, IκBβ was detected by Western blot at the indicated time points. Data are representative of three independent experiments. (**B** and **C**) B16F0 cells were untreated or treated with 14,15-EET for 48 h (B) or 10 days (C) in absence or presence of STAT3 inhibitor VIII (SI, 50 μM), SP600125 (10 μM), SB203580 (10 μM), PD98059 (10 μM), and QNZ (40 nM). The inhibitors were added on d0 (B and C) or d4 (C) respectively. The expression of *Cxcl15* gene was detected by real-time RT-PCR and ELISA. (**D**) B16F0 cells were unstimulated or stimulated with 14,15-EET. The ratios of phospho-STAT3 to STAT3 (p-STAT3/STAT3) or phospho-JNK to JNK (p-JNK/JNK) at the indicated time points were calculated after densitometric analysis of Western blots. Data are pooled from three independent experiments with a total of six samples in each group. *p < 0.05, **p < 0.01.

In different types of cells, IL-8 expression could potentially be induced or promoted by different signaling pathways, including NF-κB, ERK, p38 MAPK, JNK, and STAT3 pathways [25–28]. To identify the signaling pathway(s) required for 14,15-EET to induce hIL-8/mCXCL15 expression in tumor cells, we detected the activation of these pathways by 14,15-EET. The results showed that 14,15-EET could activate STAT3 and three MAPK pathways, but not NF-κB pathway, in B16F0 (Figure 4A) and HepG2 cells (Supplementary Figure S5A). We then stimulated these cells with 14,15-EET in presence of STAT3 inhibitor VIII, PD98059 (inhibitor of ERK pathway), SB203580 (p38 MAPK inhibitor), SP600125 (JNK inhibitor), and QNZ (NF-κB inhibitor). hIL-8/mCXCL15 expression was not significantly influenced by the inhibitors of ERK, p38 MAPK, and NF-κB pathways. Inhibiting JNK pathway suppressed both early up-regulation (first 48 h) and the continuous expression of CXCL15 in B16F0 cells (Figure 4B, 4C) and IL-8 in HepG2 cells (Supplementary Figure S5B, S5C). Consistently, the prolonged treatment with 14,15-EET induced the sustained and gradually enhanced activation of JNK pathway (Figure 4D and Supplementary Figure S5D). Differently, 14,15-EET did not induce the gradually enhanced STAT3 activation. The later inhibition of STAT3 pathway had less effect on hIL-8/mCXCL15 expression. However, the early inhibition of STAT3 pathway significantly suppressed both early up-regulation and the continuous expression of hIL-8/mCXCL15. These results suggest that the activation of STAT3 pathway might not only induce early up-regulation of hIL-8/mCXCL15 in tumor cells, but also augment the later effect of JNK pathway.

### miR-155 is required for 14,15-EET to maintain the continuous expression of hIL-8/mCXCL15

**Figure 5:**
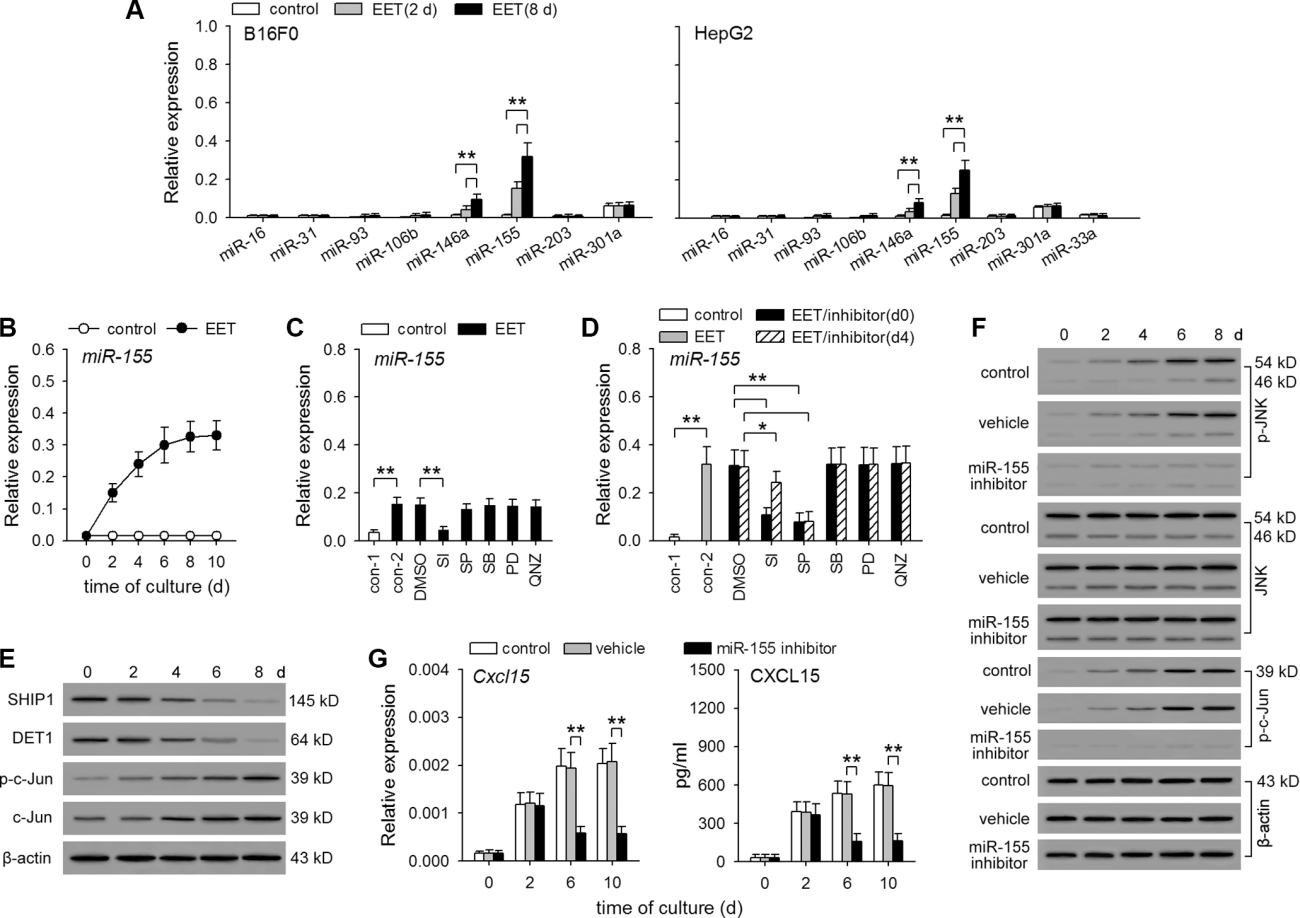
14,15-EET up-regulates miR-155 expression to maintain the sustained activation of JNK pathway (**A**) B16F0 cells and HepG2 cells were untreated or treated with 14,15-EET (100 nM) for 2 days or 8 days. The expression of the indicated microRNAs was detected by real-time RT-PCR (*n* = 6 per group). (**B** to **E**) B16F0 cells were untreated or treated with 14,15-EET. (B) The expression of miR-155 was detected by real-time RT-PCR at the indicated time points. (C and D) The cells were cultured for 48 h (C) or 10 days (D) in absence or presence of STAT3 inhibitor VIII (SI, 50 μM), SP600125 (10 μM), SB203580 (10 μM), PD98059 (10 μM), and QNZ (40 nM). The inhibitors were added on d0 (C and D) or d4 (D) respectively. The expression of miR-155 was detected by real-time RT-PCR. (E) The expression of SHIP1, DET1, c-Jun, and the phosphorylation of c-Jun, were detected by Western blot at the indicated time points. (**F** and **G**) B16F0 cells were treated with 14,15-EET in absence or presence of miR-155 inhibitor (50 nM) or vehicle. The phosphorylation of JNK and c-Jun was detected by Western blot at the indicated time points (F). The expression of *Cxcl15* gene was detected by real-time RT-PCR and ELISA at the indicated time points (G). Data are representative of three independent experiments (E, F). *p < 0.05, **p < 0.01.

To further clarify the mechanism underlying the continuous expression of hIL-8/mCXCL15 induced by 14,15-EET, we investigated whether 14,15-EET might down-regulate or up-regulate the expression of microRNAs. In different types of cells, several microRNAs have been found to influence IL-8 production. miR-93, miR-106b, and miR-203 directly bind *IL8* mRNA to suppress translation [29, 30]. miR-16, miR-31, miR-33a, miR-146a, miR-155, and miR-301a can suppress or promote IL-8 expression and secretion by inhibiting the expression of different proteins [31–36]. Among them, miR-155 was substantially up-regulated by 14,15-EET (Figure 5A). Interestingly, the prolonged treatment with 14,15-EET also induced the continuous expression of miR-155 (Figure 5B and Supplementary Figure S6A). 14,15-EET up-regulated the expression of miR-155 through STAT3 and JNK pathways (Figure 5C and 5D, and Supplementary Figure S6B, S6C). The early inhibition of STAT3 pathway significantly suppressed both early up-regulation (first 48 h) and the continuous expression of miR-155, whereas the later inhibition of STAT3 pathway had less effect on the continuous expression of miR-155. Inhibiting JNK pathway mainly suppressed the continuous expression of miR-155.

miR-155 does not directly bind IL-8 mRNA, but it can promote the activation of JNK pathway by down-regulating the expression of SHIP1 and DET1 [35, 37]. Consistent with the expression pattern of miR-155, the expression of SHIP1 and DET1 was gradually down-regulated, and the expression and activation of c-Jun was gradually augmented in B16F0 and HepG2 cells after prolonged treatment with 14,15-EET (Figure 5E and Supplementary Figure S6D). When these cells were stimulated with 14,15-EET in presence of miR-155 inhibitor, 14,15-EET did not suppress SHIP1 and DET1 expression (Supplementary Figure S6E), failed to induce the enhanced and sustained activation of JNK and c-Jun (Figure 5F and Supplementary Figure S6F), and was unable to maintain the continuous expression of CXCL15 (Figure 5G) and IL-8 (Supplementary Figure S6G). Taken together, these results suggest that 14,15-EET could up-regulate the expression of miR-155 to maintain the enhanced and sustained activation of JNK pathway, thus maintaining the continuous expression of hIL-8/mCXCL15.

### Neutrophil function is converted by 14,15-EET-treatment *in vivo*

**Figure 6:**
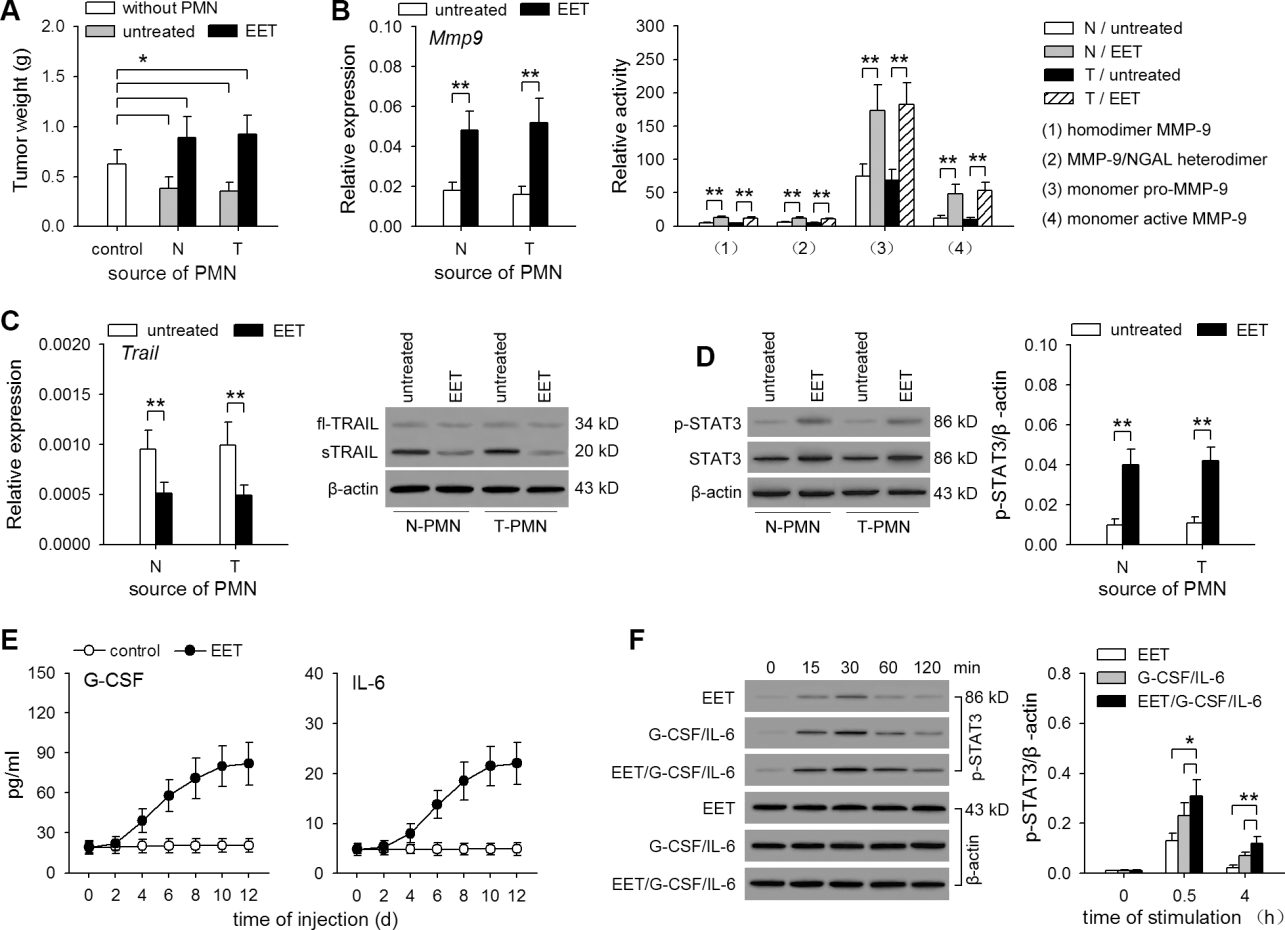
14,15-EET induces tumor-promoting the function of neutrophils (**A** to **D**) Control mice (N) and the mice inoculated with T/H/H-B16F0 cells (T) were untreated or treated with 14,15-EET. On d42 after inoculation, neutrophils were isolated from the peritoneal cavity of mice after recruitment as described in Methods. (A) Neutrophils were used for co-inoculation test as described in Methods. (B) The expression and release of MMP-9 by neutrophils were detected by real-time RT-PCR and zymography. (C) The expression of TRAIL in neutrophils was detected by real-time RT-PCR and Western blot. (D) STAT3 and phospho-STAT3 in neutrophils was detected by Western blot (left). The relative level of phospho-STAT3 to β-actin was calculated after densitometric analysis of Western blots (right). (**E**) The levels of serum G-CSF and IL-6 in naive mice and 14,15-EET-treated mice were detected by ELISA. (**F**) Neutrophils were isolated from bone marrow of naive mice, and stimulated with 14,15-EET (100 nM) and/or G-CSF/IL-6 (50 ng/ml of each). The phospho-STAT3 (p-STAT3) was analyzed at the indicated time points by Western blot (left). The relative level of p-STAT3 to β-actin was calculated after densitometric analysis of Western blots (right). Data are representative of three independent experiments (C, D, F, Western blots), or pooled from three independent experiments with a total of six samples in each group (D right, F right). *p < 0.05, **p < 0.01.

The above results indicated that 14,15-EET-induced neutrophilic infiltration could promote the growth of dormant metastases. We then further investigated the function of neutrophils in a co-inoculation test (Figure 6A) and the metastatic model using T/H/H-B16F0 or metastatic cell line B16F1 (Supplementary Figure S7A–S7E). Simply harboring dormant metastases could not induce the conversion of neutrophil function from tumor-suppressing to tumor promoting (Figure 6A). The conversion was induced by treating mice with 14,15-EET (Figure 6A, Supplementary Figure S7A–S7D), or increasing *in vivo* production of EETs with the inhibitor of soluble epoxide hydrolase (sEH, the enzyme that metabolizes 14,15-EET) (Supplementary Figure S7E). Consistently, the neutrophils isolated from 14,15-EET-treated mice, but not the mice simply harboring dormant metastases, produced more MMP-9 (Figure 6B) and expressed much less TRAIL (Figure 6C), which is in favor of angiogenesis.

We then analyzed whether 14,15-EET may influence the activation of STAT3 in neutrophils, since the enhanced activation of STAT3 results in the conversion of neutrophil function [12]. The results showed that both the expression and activation of STAT3 in neutrophils were increased by 14,15-EET-treatment *in vivo*, but not by simply harboring dormant metastases (Figure 6D and Supplementary Figure S7F). Intriguingly, the treatment with 14,15-EET, but not other regioisomers, substantially increased the serum levels of G-CSF and IL-6 (Figure 6E, and Supplementary Figure S7G). Moreover, 14,15-EET could cooperate with G-CSF/IL-6 to enhance the activation of STAT3 in neutrophils (Figure 6F), increasing the expression of *Mmp9* gene and decreasing the expression of *Trail* gene (Supplementary Figure S7H). Taken together, these results suggest that 14,15-EET could induce the enhanced activation of STAT3 in neutrophils.

### Neutrophil-derived MMP-9 is crucial for 14,15-EET to induce the growth of dormant metastases

**Figure 7:**
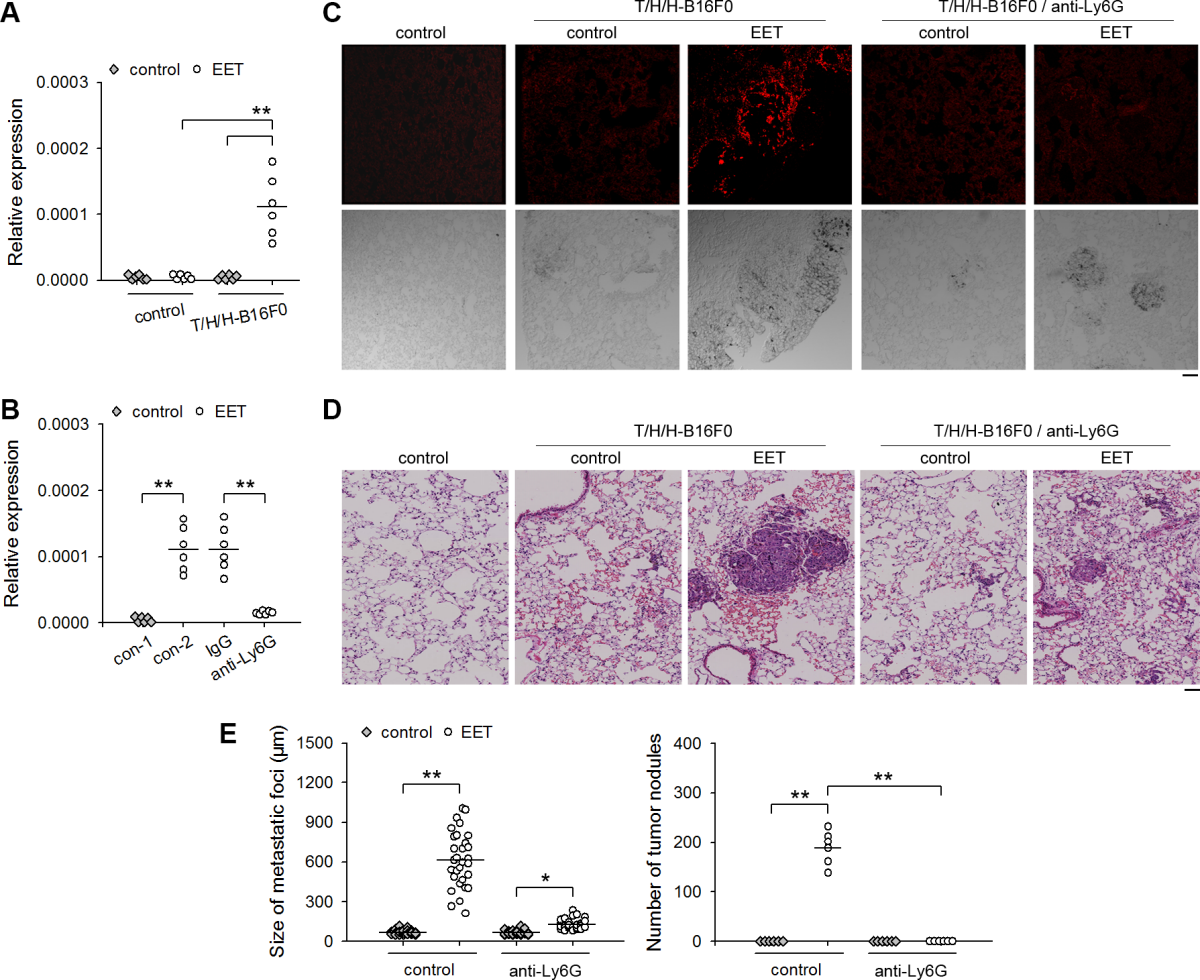
Neutrophils are the main source of MMP-9 in 14,15-EET-induced development of metastatic lesions The mice inoculated with T/H/H-B16F0 cells were untreated or treated with 14,15-EET as described in Methods. Neutrophils were not depleted (A) or depleted *in vivo* with anti-Ly6G antibody (B to E) when the mice were treated with 14,15-EET. The mice (*n* = 6 per group) were sacrificed on d42 after inoculation. (**A** and **B**) The expression of *Mmp9* gene in lung tissues was detected by real-time RT-PCR. The mice without inoculation were also used as control in A. (**C** and **D**) Lung tissue sections were stained for CD34 to identify microvessels (C), or subjected to H&E staining (D). Representative photographs are shown. Bar, 50 μm. The mice without inoculation were also used as control in C and D. (**E**) Metastatic foci in tissue sections (H&E staining) were measured (left). Metastatic nodules on the surface of lungs were counted (right). *p < 0.05, **p < 0.01.

We next investigated whether neutrophil-derived MMP-9 is crucial for 14,15-EET to trigger the growth of dormant metastases. 14,15-EET dramatically increased the level of *Mmp9* mRNA in the lung tissues harboring dormant metastases from T/H/H-B16F0 cells, but not in control lung tissues (Figure 7A). Interestingly, when mice were inoculated with T/H/H-HepG2 or T/H/H-MCF-7 cells, 14,15-EET mainly increased the levels of mouse *Mmp9* mRNA in lung tissues, but only slightly increased the levels of human *MMP9* mRNA (Supplementary Figure S8A). If neutrophils were depleted *in vivo*, 14,15-EET could not efficiently increase *Mmp9* mRNA in the lung tissues harboring metastases from B16F0, HepG2, and MCF-7 cells (Figure 7B, and Supplementary Figure S8B). These results suggested that neutrophils were the main source of MMP-9 in the lung tissues after 14,15-EET-treatment.

After inoculation with T/H/H-B16F0 cells and 14,15-EET-treatment, the angiogenesis in metastatic lesions was observed in control mice, but not neutrophil-depleted mice (Figure 7C) or MMP-9^−/−^ mice (Supplementary Figure S8C), similar to the effect of blocking VEGF *in vivo* (Supplementary Figure S8D). 14,15-EET could promote the proliferation of tumor cells (Supplementary Figure S8E and ref. 16). However, in neutrophil-depleted mice (Figure 7D, 7E, and Supplementary Figure S8F), MMP-9^−/−^ mice (Supplementary Figure S8G), and VEGF-blocking mice (Supplementary Figure S8H), 14,15-EET only slightly increased the size of microscopic metastatic foci, but did not result in the formation of macroscopic metastases. Therefore, MMP-9 from neutrophils is crucial for 14,15-EET to promote the angiogenesis in metastatic lesions and the development of metastatic lesions.

## DISCUSSION

Infiltrating neutrophils could promote tumor angiogenesis and tumor progression [6, 12]. Here, our results suggest that the inefficient recruitment of neutrophils might be an important reason that the dormancy of micrometastases could be maintained. Non-metastatic tumor cells may acquire invasive capacity in presence of TGF-β1 and neutrophil-derived H_2_O_2_ and HOCl, but the cells could only form dormant micrometastases after extravasation as shown in our present and previous studies [19]. Intriguingly, our data in this study showed that 14,15-EET could trigger the growth of dormant micrometastases by inducing neutrophil infiltration in metastatic lesions and the conversion of neutrophil function, which might be a driving force for tumor recurrence based on dormant metastases.

EETs have been known to exert antiinflammatory effects *in vivo* [14, 15]. Intriguingly, however, 14,15-EET could induce neutrophilic infiltration in metastatic lesions by activating STAT3 and JNK pathways to induce the expression of IL-8 and CXCL15 in tumor cells. Interestingly, 14,15-EET increased IL-8 expression in human tumor cells and CXCL15 expression in mouse tumor cells by activating same signaling pathways. JNK pathway can promote both expression and secretion of IL-8 [25, 38]. 14,15-EET could induce the enhanced and sustained activation of JNK pathway by increasing miR-155 expression. 14,15-EET was less efficient in inducing the transient activation of JNK pathway in tumor cells due to the inhibitory effect of SHIP1 and DET1. Therefore, although JNK pathway could promote the expression of miR-155 as shown by our data and others [39], the early up-regulation of miR-155 was mainly induced by 14,15-EET-activated STAT3. When miR-155 was up-regulated, JNK/c-Jun pathway was easier to be activated due to the down-regulation of SHIP1 and DET1 expression by miR-155. Then, the enhanced activation of JNK pathway was more efficient in maintaining the expression of miR-155, thus forming a positive feedback loop to maintain the enhanced and sustained activation of JNK pathway, and the continuous production of hIL-8/mCXCL15 that is important for the continuous recruitment of neutrophils and the development of metastatic lesions.

Although neutrophils have the potential to inhibit metastatic seeding in the premetastatic lungs [40], 14,15-EET could induce the conversion of neutrophil function *in vivo*. The enhanced activation of STAT3 in neutrophils can promote the expression and release of MMP-9, and reduce the expression of TRAIL and the release of NE [12]. The direct effect of 14,15-EET alone on neutrophils was not strong, since the activation of STAT3 in neutrophils is not as efficient as that in other types of cells [12]. The increased STAT3 expression and the co-stimulation of different factors, such as G-CSF/IL-6, are required to enhance STAT3 activation in neutrophils [12]. IL-6 can down-regulate IFN-β expression in bone-marrow to increase STAT3 expression in neutrophils [12]. G-CSF and IL-6 can potentially be produced by different cells in the setting of chronic inflammation, including endothelial cells, fibroblasts, monocytes, and other cells [41, 42]. 14,15-EET might stimulate some of these cells to increase the production of G-CSF and IL-6 *in vivo*. Moreover, 14,15-EET can cooperate with G-CSF/IL-6 to more efficiently induce the enhanced and sustained activation of STAT3, thus inducing the conversion of neutrophil function from tumor-suppressing to tumor-promoting.

All of four regioisomeric EETs have the potential to promote angiogenesis by directly stimulating endothelial cells [43]. However, only 14,15-EET could efficiently trigger the growth of dormant micrometastases as shown by our data, since only 14,15-EET could induce neutrophilic infiltration and convert neutrophil function. Neutrophils produce MMP-9 but not tissue inhibitors of metalloproteinases (TIMPs). Therefore, neutrophils release TIMP-free MMP-9 [7, 44], which is rapidly and freely available to promote angiogenesis by remodeling extracellular matrix and releasing such potent angiogenic factors as VEGF and FGF2 that are usually sequestered in an inactivated form to the ECM [7]. In contrast to neutrophils, other MMP-9-producing cells produce TIMPs, and therefore release inactive MMP-9/TIMP complexes [7, 44]. 14,15-EET could promote MMP-9 expression in tumor cells, but did not increase the production of active MMP-9 (our unpublished data). On the other hand, our data showed that neutrophils were the main source of MMP-9 in the lung tissues harboring metastases after 14,15-EET treatment. In the absence of neutrophils, 14,15-EET failed to efficiently increase *Mmp9* gene expression in the lung tissues harboring metastases. Therefore, inducing neutrophilic infiltration in metastatic lesions is crucial for 14,15-EET to activate angiogenesis and trigger the growth of dormant metastases. Thereafter, the other three regioisomeric EETs may promote the angiogenesis by stimulating endothelial cells.

14,15-EET promotes the growth of dormant micrometastases in a dose-dependent manner. Our data might have the implication that the increase of 14,15-EET *in vivo* might increase the risk of tumor recurrence based on the dormant micrometastases. EETs can be increased by factors that promote P450 expression and/or activity, or by chemicals and nutritional factors [45]. Therefore, controlling the EET levels in patients is of importance to prevent the growth of dormant micrometastases. In addition to 14,15-EET, the growth of dormant micrometastases might also be triggered by other factors through similar mechanisms. For example, infection could induce the production of G-CSF/IL-6 and PGE_2_ [46]. PGE_2_ could induce the expression of CXCL1 in tumor cells [47]. Therefore, infection-induced chronic inflammation might also promote the recruitment of tumor-promoting neutrophils to metastatic lesions. Given that there are different chemoattractants, and that protracted depletion of neutrophils is clinically untenable, exploring the approach for augmenting tumor-suppressing function of neutrophils might be very important for designing the therapeutic strategy for preventing the recurrence of tumor based on metastases.

## MATERIALS AND METHODS

### Cells and treatment

C57BL/6 background B16F0 and B16F1 melanoma cells, human hepatocellular carcinoma HepG2 cells, and human breast cancer MCF-7 cells were purchased from China Center for Type Culture Collection (CCTCC, Wuhan, China) and cultured according to their guidelines. The cell lines were authenticated at China Center for Type Culture Collection (Wuhan, China) in June 2014, using short tandem repeat (STR) DNA profiling (ABI 31300xl Genetic Analyzer; Life Technologies). To induce the invasive capacity of non-metastatic tumor cells (B16F0, HepG2, MCF-7), the cells were cultured in presence of T/H/H (TGF-β1, 5 ng/ml, H_2_O_2_, 100 μM, HOCl, 50 μM) for 10 days [19]. For the convenience of description, T/H/H-pretreated cells were mentioned as T/H/H-B16F0, T/H/H-HepG2, and T/H/H-MCF-7 cells, respectively.

### Animals

C57BL/6 mice (6–8 weeks old) were purchased from the Center of Medical Experimental Animals of Hubei Province (Wuhan, China). B6.FVB(Cg)-*Mmp9^tm1Tvu^*/J mice (MMP-9^−/−^) were kindly provided by professor Hong-Liang Li (Cardiovascular Research Institute of Wuhan University). Athymic nude (nu/nu) mice (4–5 weeks old) were purchased from Beijing HFK Bio-Technology Co. LTD. (Beijing, China). The mice were maintained in the accredited animal facility of Tongji Medical College, and animal care was in accordance with institutional guidelines. All animal experiments were approved by the Committee on the Ethics of Animal Experiments of Tongji Medical College (Permit Number: 2012-S395).

### Metastasis model and EET-treatment

To establish the model of minimal dormant metastases, C57BL/6 mice or MMP-9^−/−^ mice were inoculated with B16F0 cells (5 × 10^5^ cells/mouse), or nude mice were inoculated with HepG2 cells (4 × 10^6^ cells/mouse) or MCF-7 cells (2 × 10^6^ cells/mouse). These non-metastatic tumor cells acquired invasive capacity after pretreatment with T/H/H, and were able to extravasate after injection via tail vein (Supplementary Figure S1A and refs. 19, 20). 3 weeks after inoculation, only microscopic metastatic lesions were observed in lung tissues, and no net size increase in metastatic lesion occurred thereafter (Supplementary Figure S1B and refs. 19, 20). The dormancy of micrometastases was also shown by the reduced accumulation of Ki-67, a proliferation marker, in tumor cells (Supplementary Figure S1C and ref. 20). When indicated, the mice received the i.v. injection of 14,15-EET (30 μg/kg) and/or 14,15-EEZE (30 μg/kg) or 14,15-DHET (30 μg/kg), once every two days, from d22 to d40 after inoculation (Supplementary Figure S1D). The mice were sacrificed on the indicated time points. In B16F0-model, the black nodules, but not faint brown spots, on the surface of lung were counted as visible metastatic nodules. In HepG2- and MCF-7-model, the lungs of mice were fixed in Bouin's solution (Sigma Aldrich), displaying metastatic nodules on the surface of lung as darker color nodules.

### *In vivo* depletion of neutrophils

To deplete neutrophils, anti-Ly6G antibody was used [13]. Starting from d3 after first injection of 14,15-EET, the mice received i.p. injection of anti-Ly6G antibody at a dose of 300 μg in 500 μl PBS [48], once every three days for six times. The depletion of neutrophils was identified by flow cytometric analysis (Supplementary Figure S3B).

### Cell transfection

To inhibit the up-regulation of CXCL15 expression in B16F0 cells, the cells were transduced with sh-Cxcl15(1) and sh-Cxcl15(2) lentiviral particles (GeneChem, Shanghai, China) to express Cxcl15 shRNAs, binding 5′-CCAATTACTAACAGGTTCCTA-3′ and 5′-CCTGAGAACAAGAGAATATTT-3′, respectively. To suppress the up-regulation of IL-8 expression in HepG2 cells, the cells were transduced with sh-IL8(1) and sh-IL8(2) lentiviral particles (GeneChem) to express IL-8 shRNAs, binding 5′-CAAGGAGTGCTAAAGAACT TA-3′ and 5′-GCTCTGTGTGAAGGTGCAGTT-3′, respectively. sh-con, not binding any known mRNA, were used as control. After selection with puromycin, the cells were used for further experiments. To suppress the function of miR-155, the cells were cultured in the presence of miR-155 inhibitor/HiPerFect Transfection Reagent (Qiagen, Valencia, CA) according to the manufacturer's instructions.

### Recruitment of neutrophils to peritoneal cavity

To recruit neutrophils to peritoneal cavity, CXCL15-expressing hepatocytes were injected to peritoneal cavity of mice (3 × 10^5^ per mouse). 12 h later, the peritoneal cells were harvested for the isolation of neutrophils. To acquire CXCL15-expressing hepatocytes, mice received i.v. injection of pCxcl15 plasmid (200 μg per mouse). 12 h later, hepatocytes were prepared from liver by two-step collagenase perfusion technique [12].

### Isolation of neutrophils

Murine neutrophils were isolated from bone marrow cells or peritoneal cells as described previously [12]. Briefly, the cells were washed once in HBSS, layered over a Percoll gradient (54%/64%/72% for bone marrow cells and 54%/64%/80% for peritoneal cells), and centrifuged at 1060 × *g* for 30 min. The dense band at 64%/72% or 64%/80% interface was collected as neutrophil fraction. The isolated cells were > 90% neutrophils as assessed by flow cytometric analysis and Giemsa-Wright stain (Supplementary Figure S9).

### Co-inoculation test

Mice were inoculated intramuscularly in the right hind thigh with 3 × 10^5^ B16F0 cells, mixed with 1 × 10^6^ neutrophils. Tumors were dissected and weighed on d12 after inoculation.

### Reagents and other methods

Reagents are shown in Supplementary Material. Other methods were performed using standard protocols, including assay of tumor cell arrest in lung and extravasation, histology, ELISA analysis, Western blot assay, MMP-9 assay, immunofluorescence, analysis of gene expression and microRNA expression by real-time RT-PCR, flow cytometric analysis, soft agar assay. See Supplementary Methods for details.

### Statistical analysis

Results were expressed as mean value ± SD and interpreted by one-way ANOVA. Differences were considered statistically significant when *P* < 0.05.

## SUPPLEMENTARY MATERIAL FIGURES


