# Supplementary material for: 14,15-EET induces the infiltration and tumor-promoting function of neutrophils to trigger the growth of minimal dormant metastases
Source: Oncotarget. 2016 May 30;7(28):43324–36. doi: 10.18632/oncotarget.9709 (PMC5190026; doi:10.18632/oncotarget.9709)
Supplement: Supplementary file 1 [file oncotarget-07-43324-s001.pdf]

14,15-EET induces the infiltration and tumor-promoting function of neutrophils to trigger the growth of minimal dormant metastases

Supplementary Materials

REAGENTS

5,6-EET, 8,9-EET, 11,12-EET, 14,15-EET, 14,15-EEZE, 14,15-DHET, and *t*-AUCB were purchased from Cayman chemical. Mouse TGF-β1, human TGF-β1, mouse G-CSF and IL-6 were purchased from PeproTech (Rocky Hill, NJ). H_2O_2 and HOCl were purchased from Sigma-Aldrich (St. Louis, MO). 6-amino-4-(4-phenoxyphenylethylamino)quinazoline (QNZ), SB203580, PD98059, SP600125, and STAT3 inhibitor VIII were purchased from Merck4Biosciences (Calbiochem). All inhibitors were dissolved in DMSO as a stock solution and diluted with culture medium to the desired concentration without toxicity to cells.

SUPPLEMENTARY METHODS

Assay of tumor cell arrest in lung and extravasation

B16F0 cells, untreated or treated with T/H/H for 10 days, were labeled with CFSE. 5×10^5 CFSE-labeled cells were injected into mice via tail vein. Lungs were harvested from mice 5 h and 24 h after tumor cell injection. Frozen sections were prepared and analyzed by fluorescence microscopy. Fluorescent spots were counted from randomly chosen fields in the sections of each mouse.

Histology

Mice were anesthetized. Left ventricle was intubated and rapidly perfused with 20 ml of 0.9% saline via ascending aorta, and then continuously perfused with 30 ml paraformaldehyde with the concentration of 40 g/L. The lung tissues were harvested and embedded in paraffin according to standard histological procedures. Tissue sections were prepared and subjected to H&E staining or immunohistochemical analysis. For H&E staining, the sections were stained with hematoxylin and eosin. 5 metastatic foci in each mouse were randomly chosen for measuring the size, which was calculated using the formula: (length + width)/2. Immunohistochemical staining for proliferation marker Ki-67 was performed using anti-mouse Ki-67 antibody (Abcam Biotechnology) as primary antibody. HRP-conjugated anti-rabbit IgG was used as secondary antibody. Images were obtained using OLYMPUS-BX51 microscope at 10 × 10 or 40 × 10 magnification. Staining intensity of cells was evaluated under a microscope and graded (1, weak; 2, moderate; 3, strong) in a double blinded fashion (stainer- and examiner-blind). Staining intensity of tissue sections was assessed using a semi-quantitative immunohistochemical scoring system, HSCORE. The HSCORE was calculated using the following equation: HSCORE = $\sum Pi(i + 1)$, where i is the staining intensity of cells and Pi is the percentage of the cells at each level of intensity [20].

For detecting neutrophils, anti-mouse Ly6G antibody (Santa Cruz Biotechnology, Santa Cruz, CA) and HRP-conjugated secondary antibody were used for immunohistochemical analysis. Images were obtained using OLYMPUS-BX51 microscope at 10 × 10 and 40 × 10 magnification. The neutrophils were counted by using Image-pro-plus 6.0 software. The neutrophil density was defined as the number of neutrophils per microscopic field with metastatic lesion.

ELISA analysis

Cell-free supernatants from untreated or 14,15-EET-treated tumor cells were harvested at the indicated time points. hIL-8/mCXCL15 in the supernatants was quantified using human IL-8 and mouse CXCL15 ELISA kit (R&D Systems, Minneapolis, MN) according to the manufacturer's protocol. To determine the effect of 14,15-EET on the production of G-CSF and IL-6 *in vivo*, mice were treated with 14,15-EET (30 μg/kg) by i.v. injection, once every two days. Serum levels of G-CSF and IL-6 were detected using mouse G-CSF and IL-6 ELISA kits (R&D Systems, Minneapolis, MN).

Western blot assay

Cells were treated with the indicated stimuli or isolated from mice. Western blot assay was done as described previously [49, 50]. Primary antibodies and horseradish peroxidase-conjugated secondary antibodies were purchased from Santa Cruz Biotechnology (Santa Cruz, CA), R&D systems (Minneapolis, CA), and Cell Signaling (Beverly, MA), respectively.

MMP-9 assay

To detect neutrophil-released MMP-9, neutrophils were incubated at the concentration of 5×10^6/ml at 37 °C for 4 h in RPMI 1640 medium [12]. MMP-9 in

supernatants was detected by gelatin zymography, and the relative activity of MMP-9 was calculated as described previously [49].

Immunofluorescence

The lung tissues were harvested at the indicated time points after tumor cell inoculation. Frozen tissue sections were prepared and subjected to immunofluorescence analysis as previously described [49]. For detecting neutrophils, anti-mouse Ly6G antibody (Santa Cruz Biotechnology) was used as primary antibody. The sections were further stained with Cy3-conjugated secondary antibody. For detecting microvessels, anti-mouse CD34 antibody (Santa Cruz Biotechnology) and Cy3-conjugated secondary antibody were used. Images were obtained using a laser scanning confocal microscope (Olympus, FV500, Japan).

Analysis of gene expression by real-time RT-PCR

Total RNA was extracted from cells with TRIzol reagent (Invitrogen) or lung tissues homogenized in TRIzol according to the manufacturer's instructions. For real-time RT-PCR assays, the cDNA sequences of all detected genes were retrieved from NCBI database. The primers were designed with the Oligo Primer Analysis 4.0 software and the sequences were blasted (http://blast.ncbi.nlm.nih.gov/Blast.cgi). 100 ng of total RNA was used for reverse transcription using Superscript II RNase H reverse transcriptase (Invitrogen) in a volume of 25 µl. Then 2 µl of cDNA was amplified with SYBR Green Universal PCR Mastermix (Bio-Rad, Richmond, CA) in duplicate. For sample analysis, the threshold was set based on the exponential phase of products, and C_T value for samples was determined. The resulting data were analyzed with the comparative C_T method for relative gene expression quantification against house keeping gene *Gapdh*(m) or *GAPDH*(h).

The sequences of the primers used for detecting gene expression were as follows: *Mmp9*(m), sense 5′-AG TGGGACCATCATAACATCACAT-3′, antisense 5′-TCT CGCGGCAAGTCTTCAG-3′; *Trail*(m), sense 5′-TACTGG GATCACTCGGAGAAG-3′, antisense 5′-A CGTGGTT GAGAAATGAATGCC-3′; *Itgam*(m), sense 5′-GATGCT TACCTGGGTTATGCT TCT-3′, antisense 5′-CCGAGGTG CTCCTAAAACCA-3′; *Ly6g*(m), sense 5′-CCATCTGC CC CACTACTC-3′, anti-sense 5′-CTGAACAGAAGCACCC CT-3′; *Cxcl1*(m), sense 5′-ACC CAAACCGAAGTCATA GC-3′, antisense 5′-AGAAGCCAGCGTTCACCAGA-3′; *Cxcl2*(m), sense 5′-CCCAGACAGAAGTCATAGC-3′, antisense 5′-TCCTTTCCAGGTCAGTTAG-3′; *Cxcl5*(m), sense 5′-CTGGCATTTCTGTTGCTGTT-3′, antisense 5′-TATGACTTCCACCG TAGGGC-3′; *Cxcl15*(m), sense 5′-TATTCCCGCGTTAGTCTGGTG-3′, antisense 5′-GC CCATAGTGGAGTGGGATAAG-3′; *Ccl2*(m), sense 5′-TG GGTCCAGACATACATT-3′, antisense 5′-ACGGGTCAA CTTCACATT-3′; *Ccl3*(m), sense 5′-ATTCCACGCCAA TTCAT C-3′, antisense 5′-GCATTCAGTTCCAGGT CA-3′; *Ccl4*(m), sense 5′-TTGCTCGTGGCT GCCTT CT-3′, antisense 5′-ACTGCTGGTCTCATAGTAATC-3′; *Ccl5*(m), sense 5′-ACCA CTCCCTGCTGCTTT-3′, antisense 5′-ACACTTGGCGGTTCCTTC-3′; *Csf2*(m), sense 5′-T CGAGCAGGGTCTACGGGGC-3′, antisense 5′-TCCGTTTCCGGAGTTGGGGG-3′; *Stat3*(m), sense 5′-ACCTCCAGGACGACTTTGAT-3′, antisense 5′-TGT CTTCTGCACGT ACTCCA-3′; *Gapdh*(m), sense 5′-ATGT TCCAGTATGACTCCACTCAC-3′, antisense 5′-GACA CCAGTAGACTCCACGACATA-3′; *MMP9*(h), sense 5′-CAGTCCACCCTTGT GCTCTTCC-3′, antisense 5′-CTGCCACCCGAGTGTAACCAT-3′; *CXCL8*(h)(*IL8*), sense 5′-GAATTCTCAGCCCTCTTCAAAAAC-3′, antisense 5′-GCCAAGGAGTGCTAAAGA ACTTAG-3′; *GAPDH*(h), sense 5′-TCATTGACTCAACTACATGGT TT-3′, antisense 5′-GAAGATGGT GATGGGATTTC-3′.

Flow cytometric analysis

To analyze the effect of neutrophil depletion *in vivo*, mice were sacrificed one day after the second injection and the last injection of anti-Ly6G antibody. The heparinized blood was harvested for analysis. 20 µl of whole blood was incubated with PE-Cy7-anti-mouse CD11b, PE-anti-mouse Ly6G or PE-anti-mouse Ly6C, APC-anti-mouse Gr-1, and FITC-anti-mouse F4/80 (eBioscience) for 30 min on ice. RBCs were then lysed. The samples were centrifuged at 350 × *g* for 5 min, and then resuspended in 300 µl of PBS for flow cytometric analysis. $CD11b^+Ly6G^+$ cells were considered as neutrophils.

When neutrophils were isolated using Percoll gradient, the isolated cells were assessed by flow cytometric analysis using PE-Cy7-anti-mouse CD11b and PE-anti-mouse Ly6G or PE-anti-mouse Ly6C antibodies (eBioscience).

Analysis of microRNA expression by real-time RT-PCR

Total RNA was extracted from cells with TRIzol reagent (Invitrogen). The relative quantity of microRNAs was determined by real-time RT-PCR. The resulting data were analyzed with the comparative C_T method for relative microRNA expression quantification against house keeping gene *Gapdh*(m) or *GAPDH*(h).

To detect the expression of microRNAs, the primers with stem-loop structure were used for reverse transcription. A microRNA universal primer and the specific sense primers were used for PCR amplification. The sequences of the primers used for detecting microRNA expression were as follows: microRNA universal primer, 5′-GACTGTTCCTCTCTTCCTC -3′; miR-16, reverse 5′-GCGACTGTTCCTCTCTTCCTCTGTGTGTGTGTG TGTAGTCG CAACGCCAAT-3′, sense 5′-GGGTAGCA GCACGTAAAT-3′; miR-31, reverse(m) 5′-GCG ACTGTTCCTCTCTTCCTCTGTGTGTGTGTGTGTAG TCGCAACAGCTAT-3′, reverse(h) 5′-GCGACTGTTCC

TCTCTTCCTCTGTGTGTGTGTGTGTAGTCGCAAA GCTATG-3′, sense 5′-GGGAGGCAAGATGCTGG-3′, miR-33a, reverse 5′-GCGACTGTTCCTCTCTT CCTCT GTGTGTGTGTGTGTGTAGTCGCAATGCAATG-3′, sense 5′-GCCCGTGCATTGTA GTTG-3′; miR-93: reverse 5′-GC GACTGTTCCTCTCTTCCTCTGTGTGTGTGTGTGTA G TCGCAACTACCTG-3′, sense 5′-GGCAAAGTGCTGT TCGTG-3′; miR-106b, reverse 5′-G CGACTGTTCCTC TCTTCCTCTGTGTGTGTGTGTGTAGTCGCAAATCT GCA-3′, sense 5′-GCCCTAAAGTGCTGACAG-3′; miR-146a: reverse 5′-GCGACTGTTCCTCTCTTCC TCTGTG TGTGTGTGTGTAGTCGCAAAACCCAT-3′, sense 5′-GG CTGAGAACTGAATT CC-3′; miR-155, reverse 5′-GCG ACTGTTCCTCTCTTCCTCTGTGTGTGTGTGTGTAG TCGCAAACCCCTA-3′, sense(m) 5′-GCTTAATGCTA ATTGTGA-3′, sense(h) 5′-GCTTAA TGCTAATCGT GA-3′; miR-203, reverse 5′-GCGACTGTTCCTCTCTTCC TCTGTGTGT GTGTGTGTAGTCGCAACTAGTG G-3′, sense 5′-GGGGTGAAATGTTTAGGA-3′; miR-301a, reverse 5′-GCGACTGTTCCTCTCTTCCTCTGTGT GTGTGTGTGTAGTCGCA AAGTAGTG-3′, sense 5′-GGGGCTCTGACTTTATTG-3′.

Soft agar assay

Tumor cells were pretreated with 14,15-EET (100 nM) for 10 days. The cells were then suspended in 0.3% agar in DMEM (20% FBS) and plated (1×10^4 cells/well in 6-well plates) on a layer of 0.6% agar in DMEM (20% FBS) in triplicate. After 21-day culture in the presence of 14,15-EET, the colonies of tumor cells were photographed under a microscope.

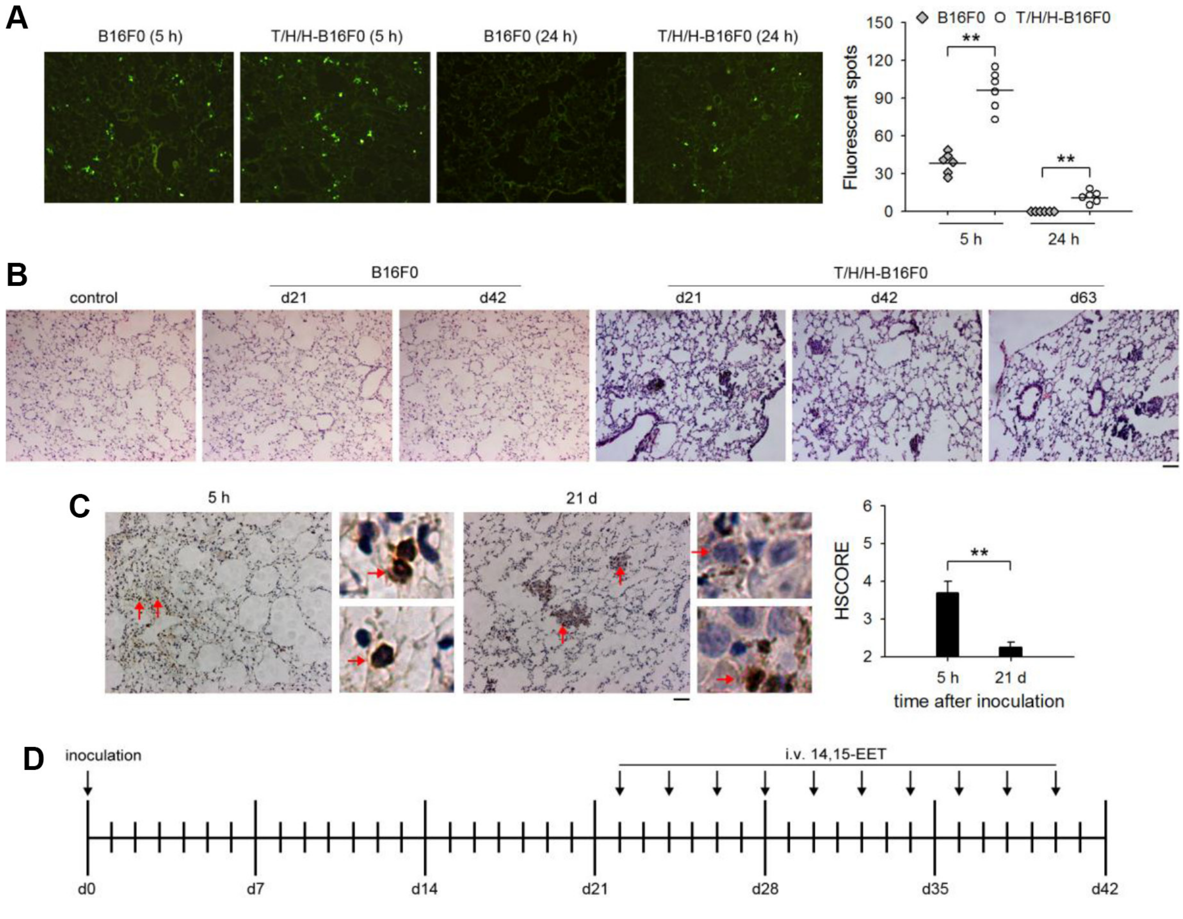

Supplementary Figure S1: The metastatic model and 14,15-EET treatment. (**A**) Tumor cell arrest in lung and extravasation. B16F0 cells and T/H/H-B16F0 cells were labeled with CFSE, and then injected to mice via tail vein (5×10^5 cells/mouse). Mice were sacrificed 5 h (for analysis of tumor cell arrest) and 24 h (for analysis of extravasation) after the i.v injection of CFSE-labeled cells. The CFSE-labeled cells in frozen sections were visualized by fluorescence microscopy (left). Fluorescent spots in the frozen sections of lung tissues were counted (right). (**B**) B16F0 cells or T/H/H-B16F0 cells were inoculated to mice via tail vein. The mice ($n = 6$ per group) were sacrificed at the indicated time points after inoculation. The sections of lung tissues were prepared and subjected to H&E staining. The mice without inoculation were used as control. Bar, 50 µm. (**C**) T/H/H-B16F0 cells were inoculated to mice via tail vein. The mice ($n = 6$ per group) were sacrificed 5 h or 21 days after inoculation. The sections of lung tissues were prepared and stained with anti-mouse Ki-67 antibody (left). In each photograph, 2 representative sites (indicated by arrows) were further amplified to show the expression of Ki-67 in tumor cells (indicated by arrows). Bar, 50 µm. The staining intensity of Ki-67 was measured using the HSCORE scoring system (right) as described in Methods. (**D**) The protocol of EET-treatment. After the inoculation of tumor cells via tail vein, the mice received i.v. injection of 14,15-EET via tail vein at the indicated time points. $^{**}p < 0.01$.

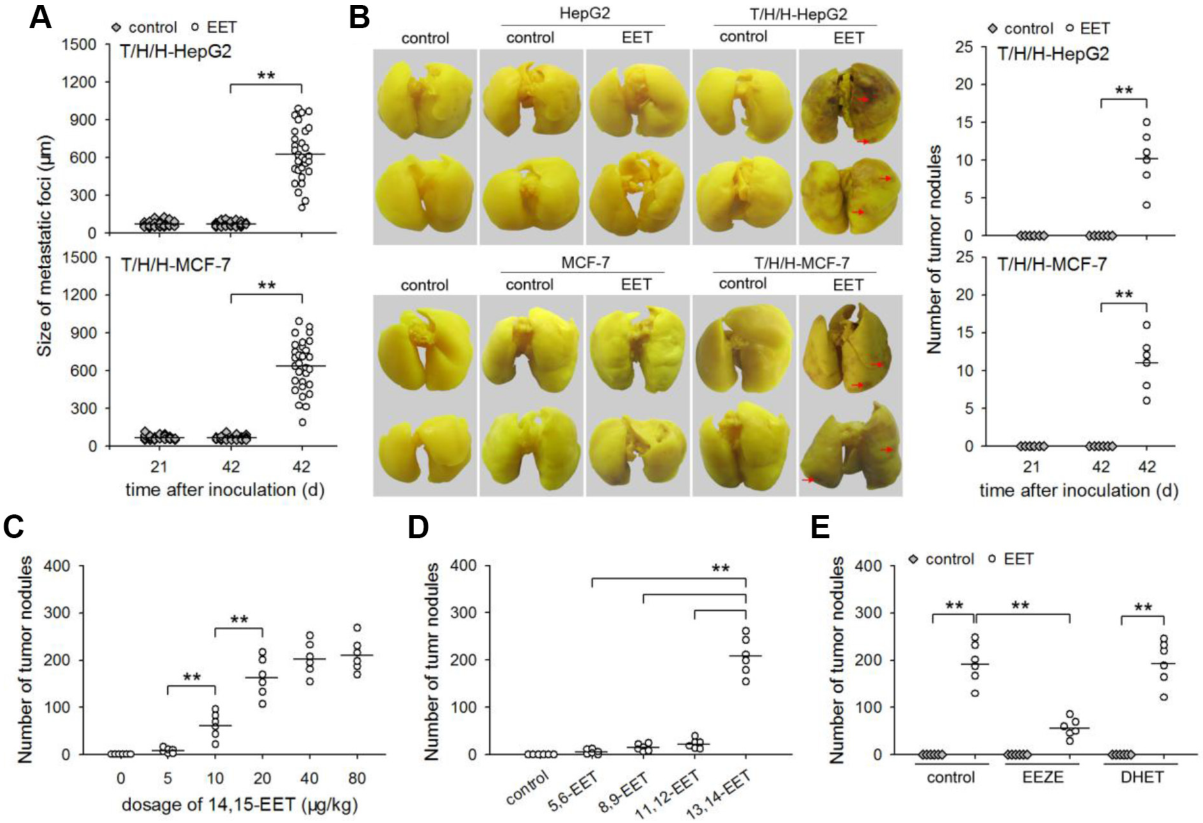

Supplementary Figure S2: The effect of 14,15-EET on the development of micrometastases. (**A** and **B**) HepG2 cells and MCF-7 cells, untreated or pretreated with T/H/H, were injected to mice via tail vein. The mice (*n* = 6 per group) were untreated or treated with 14,15-EET, and were sacrificed at the indicated time points or on d42 (B, left) after inoculation. (A) The sections of lung tissues were prepared and subjected to H&E staining. The size of the metastatic foci was measured as described in Methods. (B) The mice without inoculation were also used as control (left). The visible metastatic nodules on the surface of lung were displayed (left) and counted (right). (**C**) T/H/H-B16F0 cells were injected to mice via tail vein. The mice (*n* = 6 per group) were untreated or treated with different dosage of 14,15-EET, and were sacrificed on d42 after inoculation. Metastatic nodules on the surface of lungs were counted. (**D**) T/H/H-B16F0 cells were injected to mice via tail vein. The mice (*n* = 6 per group) were untreated or treated with 5,6-EET, 8,9-EET, 11,12-EET, and 14,15-EET (30 μg/kg) according to the protocol shown in Fig. S1D. The mice were sacrificed on d42 after inoculation. Metastatic nodules on the surface of lungs were counted. (**E**) Mice inoculated with T/H/H-B16F0 cells were untreated or treated with 14,15-EET and/or 14,15-EEZE or 14,15-DHET. The mice were sacrificed on d42 after inoculation. Metastatic nodules on the surface of lungs were counted. $^{**}p < 0.01$.

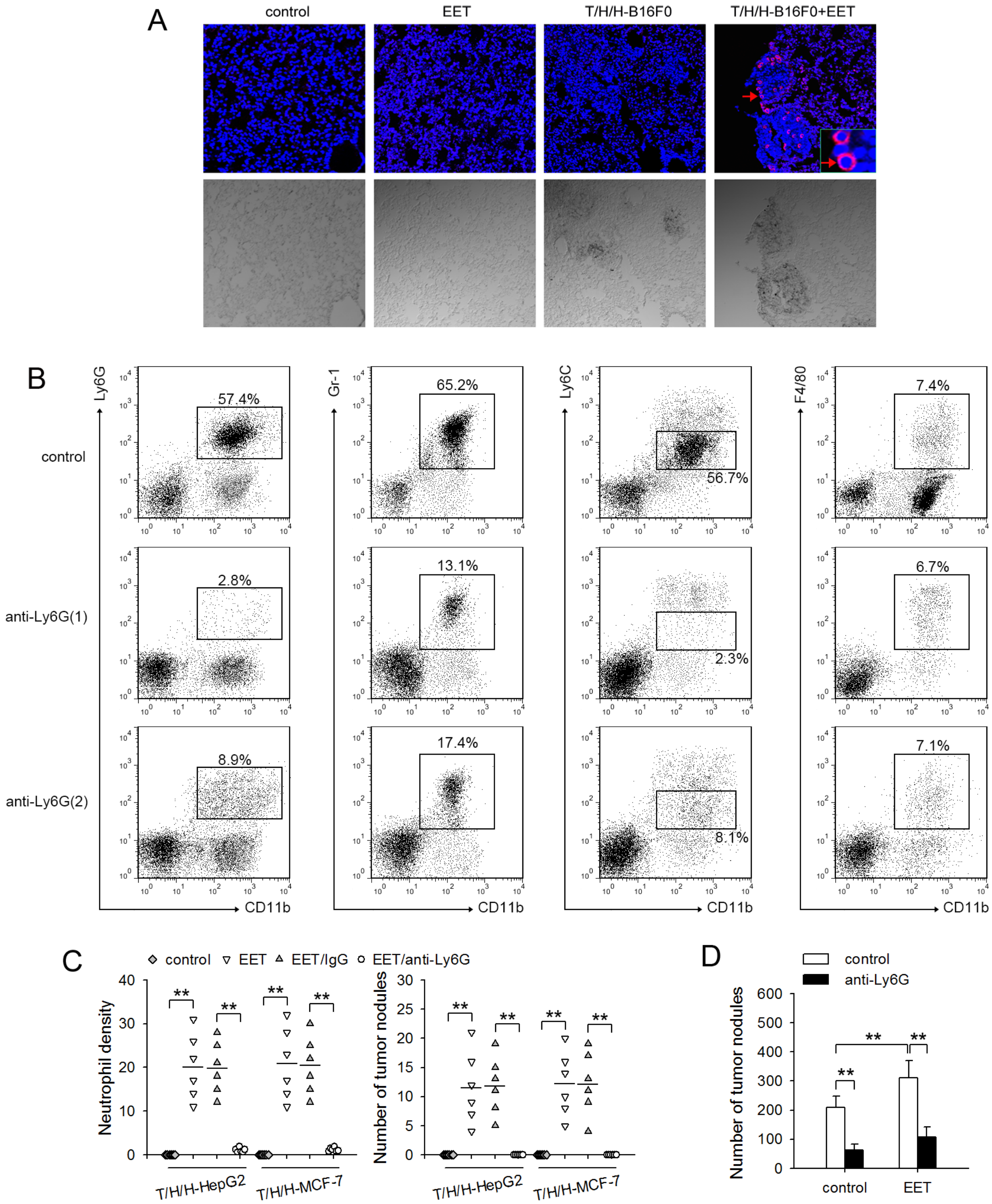

Supplementary Figure S3: Neutrophils are required for 14,15-EET to promote the development of micrometastases. (**A**) Control mice and the mice inoculated with T/H/H-B16F0 cells were untreated or treated with 14,15-EET. The mice were sacrificed on d42 after inoculation. The frozen sections of lung tissues were prepared and subjected to immunofluorescence analysis. The sections were stained for Ly6G (red) to identify neutrophils. Cell nuclei were stained with 4′,6-diamidino-2-phenylindole (DAPI, blue). Insets are the high-power view of neutrophils in corresponding picture indicated by arrow. (**B**) Anti-Ly6G antibody was used to deplete neutrophils *in vivo* as described in Methods. The neutrophils ($CD11b^+Ly6G^+$) in blood were detected by flow cytometry one day after the second injection (anti-Ly6G(1)) and last injection (anti-Ly6G(2)) of the antibody. The cells were also analyzed by staining for Ly6C. The monocytes ($F4/80^+$) were also analyzed. (**C**) Mice inoculated with T/H/H-HepG2 or T/H/H-MCF-7 cells were untreated or treated with 14,15-EET. Anti-Ly6G antibody was used to deplete neutrophils *in vivo* when the mice were treated with 14,15-EET. The mice ($n = 6$ per group) were sacrificed on d42 after inoculation. Neutrophil density in lung tissue sections was determined after immunofluorescence staining (left). Metastatic nodules on the surface of lungs were counted (right). (**D**) Mice were inoculated with B16F1 cells (5×10^5 cells/mouse). The mice received the i.v. injection of 14,15-EET (30 μg/kg), once every two days, form d1 to d19 after inoculation. Anti-Ly6G antibody was used to deplete neutrophils *in vivo* from d8 to d20 after inoculation. The mice ($n = 6$ per group) were sacrificed on d21 after inoculation. Metastatic nodules on the surface of lungs were counted. $*p < 0.05$, $**p < 0.01$.

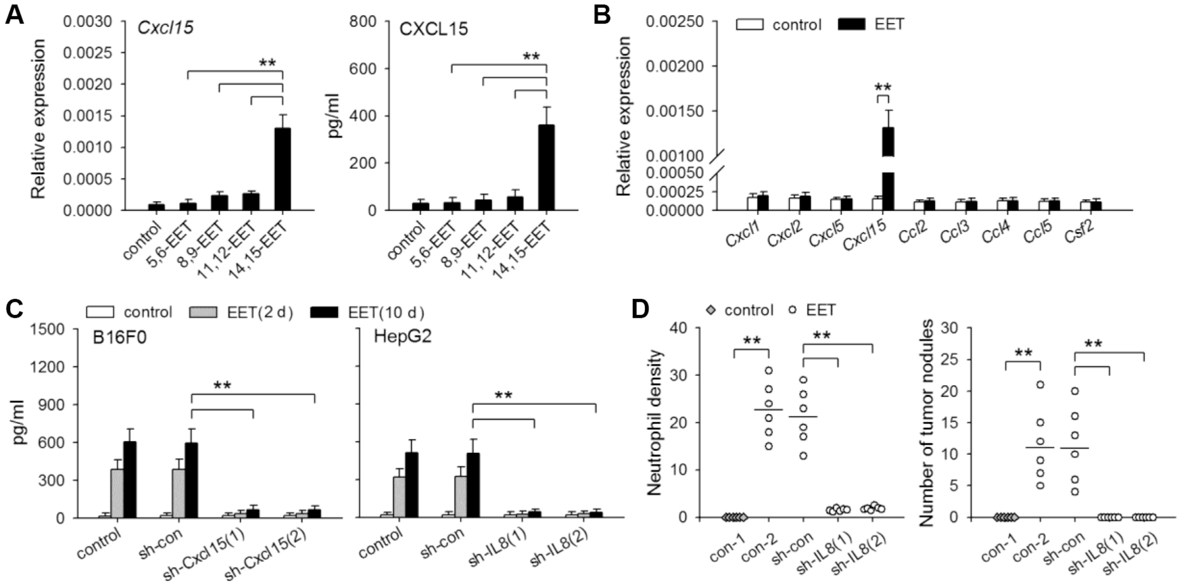

Supplementary Figure S4: 14,15-EET-induced hIL-8/mCXCL15 expression in tumor cells is required for the development of micrometastases. (**A**) B16F0 cells were untreated or treated for 48 h with 5,6-EET, 8,9-EET, 11,12-EE, and 14,15-EET (100 nM). The expression of *Cxcl15* gene was detected by real-time RT-PCR and ELISA ($n = 6$ per group). (**B**) B16F0 cells were untreated or treated with 14,15-EET (100 nM) for 48 h. The mRNA levels of the indicated mouse genes were detected by real-time RT-PCR ($n = 6$ per group). (**C**) B16F0 cells and HepG2 cells, untransfected or transfected with the indicated vectors, were untreated or treated with 14,15-EET (100 nM) for 2 days or 10 days. The expression of CXCL15 (B16F0 cells) and IL-8 (HepG2 cells) was detected by ELISA ($n = 6$ per group). (**D**) HepG2 cells, untransfected or transfected with the indicated vectors, were injected to mice ($n = 6$ per group) via tail vein after T/H/H treatment. The mice were untreated or treated with 14,15-EET, and were sacrificed on d42 after inoculation. Neutrophil density in lung tissue sections was determined after immunofluorescence staining (left). Metastatic nodules on the surface of lungs were counted (right). $^{**}p < 0.01$.

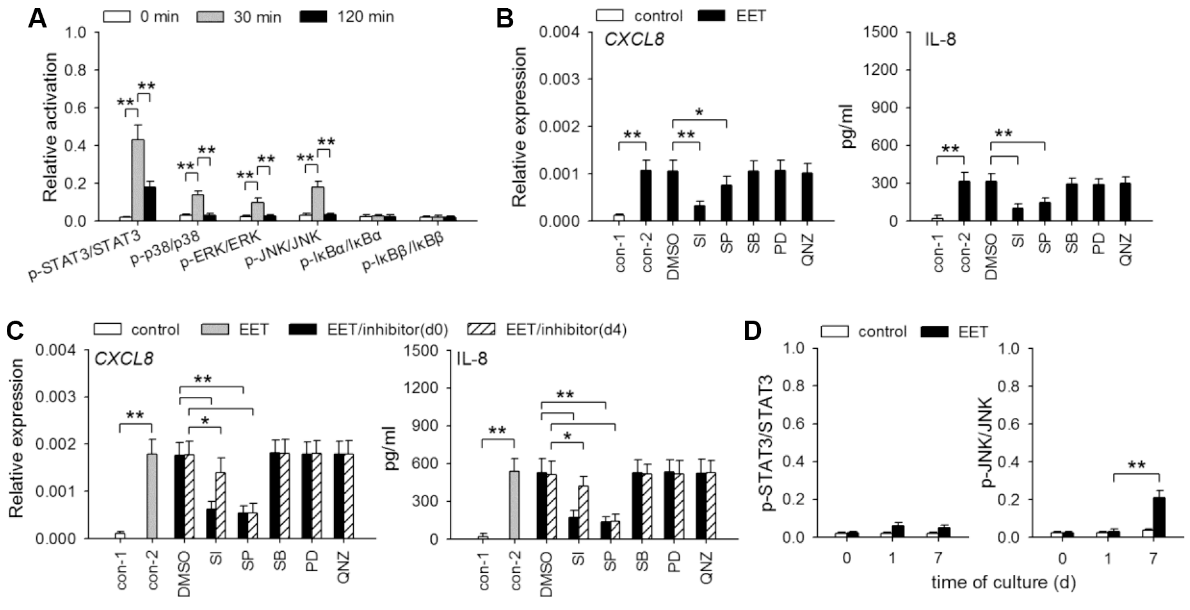

Supplementary Figure S5: 14,15-EET induces IL-8 expression by activating STAT3 and JNK pathways. (**A**) HepG2 cells were stimulated with 14,15-EET (100 nM). The phosphorylation of STAT3, p38 MAPK, ERK, JNK, IκBα, IκBβ was detected by Western blot at the indicated time points. The ratios of phospho-STAT3 to STAT3 (p-STAT3/STAT3), phospho-p38 MAPK to p38 MAPK (p-p38/p38), phospho-ERK to ERK (p-ERK/ERK), phospho-JNK to JNK (p-JNK/JNK), phospho-IκBα to IκBα (p-IκBα/IκBα), and phospho-IκBβ to IκBβ (p-IκBβ/IκBβ) were calculated after densitometric analysis of Western blots. Data are pooled from three independent experiments with a total of six samples in each group. (**B** and **C**) HepG2 cells were untreated or treated with 14,15-EET for 48 h (B) or 10 days (C) in absence or presence of STAT3 inhibitor VIII (SI, 50 μM), SP600125 (10 μM), SB203580 (10 μM), PD98059 (10 μM), and QNZ (40 nM). The inhibitors were added on d0 (B and C) or d4 (C) respectively. The expression of *CXCL8* (*IL8*) gene was detected by real-time RT-PCR and ELISA. $n = 6$ per group. (**D**) HepG2 cells were unstimulated or stimulated with 14,15-EET. The ratios of phospho-STAT3 to STAT3 (p-STAT3/STAT3) or phospho-JNK to JNK (p-JNK/JNK) at the indicated time points were calculated after densitometric analysis of Western blots. Data are pooled from three independent experiments with a total of six samples in each group. $^{**}p < 0.01$.

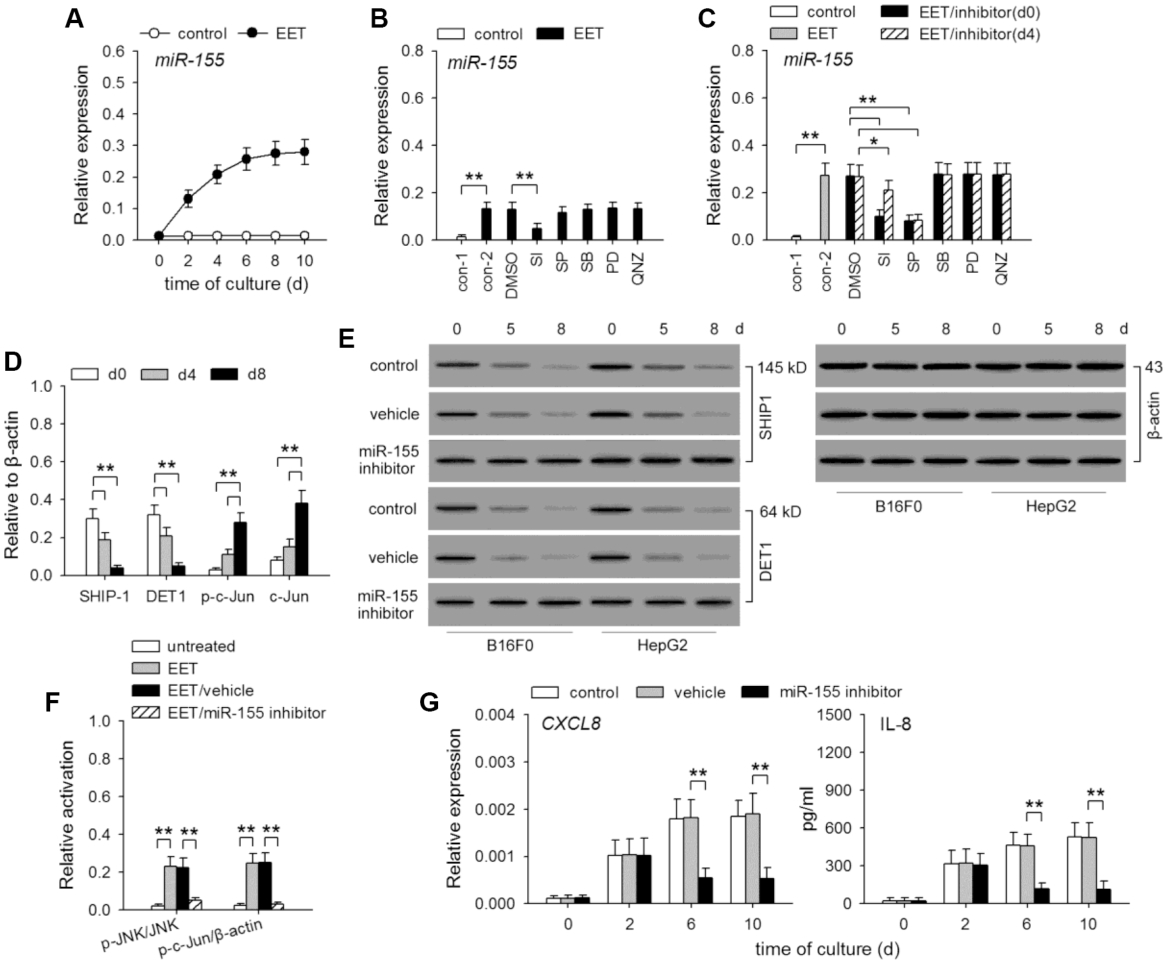

Supplementary Figure S6: miR-155 is required for 14,15-EET to maintain continuous expression of IL-8. (**A**) HepG2 cells were untreated or treated with 14,15-EET (100 nM). The expression of miR-155 was detected by real-time RT-PCR at the indicated time points (n = 6 per group). (**B** and **C**) HepG2 cells were untreated or treated with 14,15-EET for 48 h (B) or 10 days (C) in absence or presence of STAT3 inhibitor VIII (SI, 50 μM), SP600125 (10 μM), SB203580 (10 μM), PD98059 (10 μM), and QNZ (40 nM). The inhibitors were added on d0 (B and C) or d4 (C) respectively. The expression of miR-155 was detected by real-time RT-PCR. n = 6 per group. (**D**) HepG2 cells were treated with 14,15-EET. The expression of SHIP1, DET1, c-Jun, and the phosphorylation of c-Jun, were detected by Western blot at the indicated time points. The levels of SHIP1, DET1, p-c-Jun, and c-Jun relative to β-actin were calculated after densitometric analysis of Western blots. Data are pooled from three independent experiments with a total of six samples in each group. (**E**) B16F0 cells and HepG2 cells were treated with 14,15-EET in absence or presence of miR-155 inhibitor (50 nM) or vehicle. The expression of SHIP1 and DET1 was detected by Western blot at the indicated time points. Data are representative of three independent experiments. (**F**) HepG2 cells were untreated or treated with 14,15-EET for 8 days in absence or presence of miR-155 inhibitor or vehicle. The phosphorylation of JNK and c-Jun was detected. The ratios of phospho-JNK to JNK (p-JNK/JNK) and phospho-c-Jun to β-actin (p-c-Jun/β-actin) were calculated after densitometric analysis of Western blots (n = 6 per group). (**G**) HepG2 cells were treated with 14,15-EET in absence or presence of miR-155 inhibitor or vehicle. The expression of *CXCL8* (*IL8*) gene was detected by real-time RT-PCR and ELISA at the indicated time points (n = 6 per group). *$p < 0.05$, **$p < 0.01$.

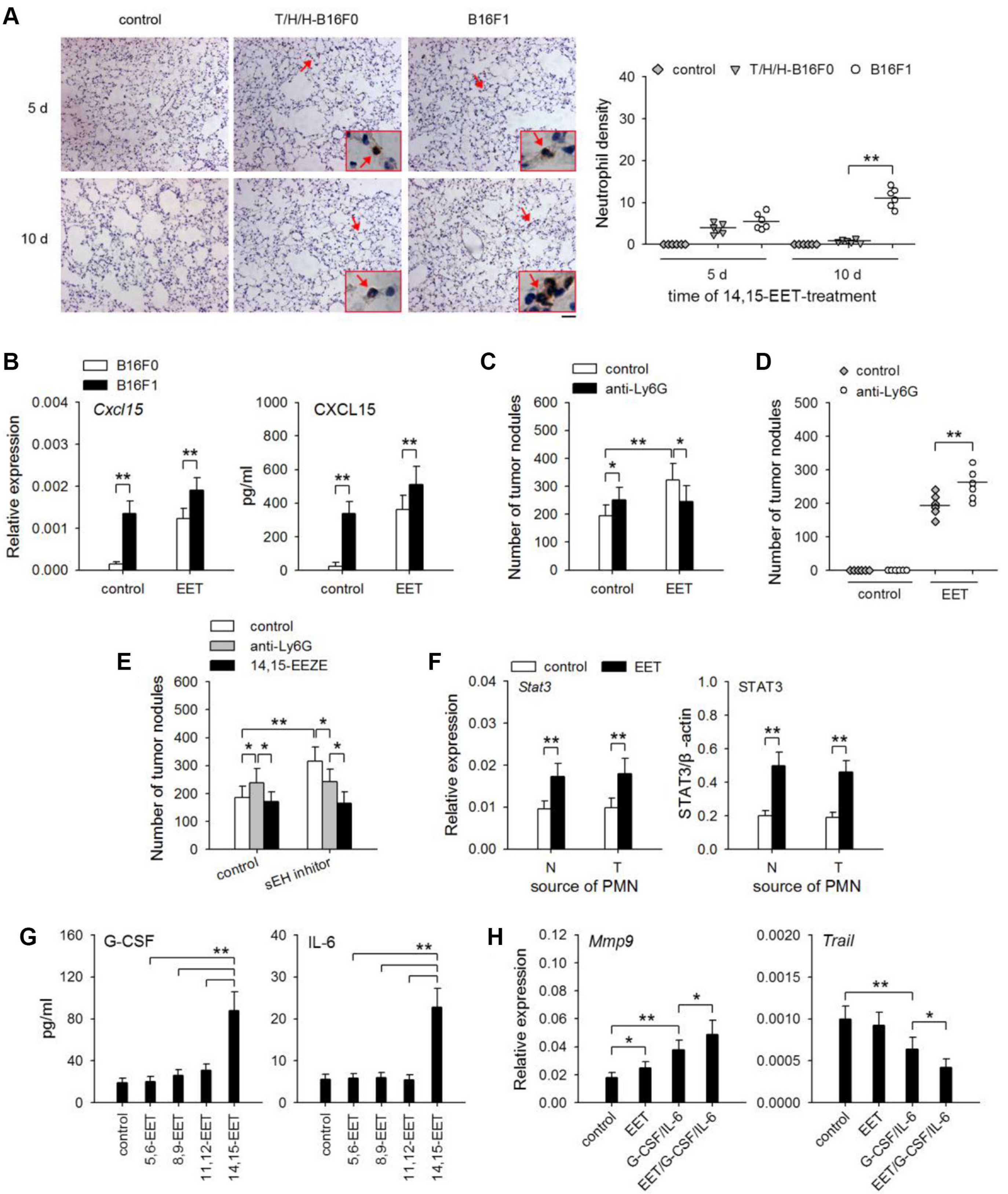

A
control
T/H/H-B16F0
B16F1
5 d
10 d
control
T/H/H-B16F0
B16F1
Neutrophil density
40
30
20
10
0
**
5 d
10 d
time of 14,15-EET-treatment
B
B16F0
B16F1
Cxcl15
Relative expression
0.004
0.003
0.002
0.001
0.000
**
**
control
EET
CXCL15
pg/ml
1000
800
600
400
200
0
**
**
control
EET
C
control
anti-Ly6G
Number of tumor nodules
600
500
400
300
200
100
0
**
*
*
control
EET
D
control
anti-Ly6G
Number of tumor nodules
500
400
300
200
100
0
**
control
EET
E
control
anti-Ly6G
14,15-EEZE
Number of tumor nodules
600
500
400
300
200
100
0
**
*
*
*
*
*
control
sEH inhitor
F
control
EET
Stat3
Relative expression
0.04
0.03
0.02
0.01
0.00
**
**
N
T
source of PMN
STAT3
STAT3/β -actin
1.0
0.8
0.6
0.4
0.2
0.0
**
**
N
T
source of PMN
G
G-CSF
pg/ml
160
120
80
40
0
**
control
5,6-EET
8,9-EET
11,12-EET
14,15-EET
IL-6
40
30
20
10
0
**
control
5,6-EET
8,9-EET
11,12-EET
14,15-EET
H
Mmp9
Relative expression
0.12
0.10
0.08
0.06
0.04
0.02
0.00
*
**
*
control
EET
G-CSF/IL-6
EET/G-CSF/IL-6
Trail
0.0020
0.0015
0.0010
0.0005
0.0000
**
*
control
EET
G-CSF/IL-6
EET/G-CSF/IL-6

Supplementary Figure S7: 14,15-EET induces the conversion of neutrophil function from tumor-suppressing to tumor-promoting. (**A**) Neutrophils were continuously recruited by B16F1 cells, but not T/H/H-B16F0 cells, at the early stage of metastasis. T/H/H-B16F0 cells (5×10^5 cells/mouse) or B16F1 cells (5×10^5 cells/mouse) were inoculated to mice by injection via tail vein. The mice ($n = 6$ per group) were sacrificed on d5 and d10 after inoculation. The sections of lung tissues were subjected to immunohistochemical staining for identifying the infiltration of neutrophils (left, Bar, 50 μm). Neutrophil density in lung tissue sections was determined after immunohistochemical staining (right). (**B**) The expression of CXCL15 in B16F1 cells is much higher than that in B16F0 cells. B16F0 cells and B16F1 cells were untreated or treated with 14,15-EET (100 nM) for 48 h. The expression of *Cxcl15* gene was detected by real-time RT-PCR and ELISA. (**C**) 14,15-EET induces the conversion of neutrophil function *in vivo*. Mice were inoculated with B16F1 cells (5×10^5/mouse) by injection via tail vein. The mice received the i.v. injection of 14,15-EET (30 μg/kg), once every two days, form -d10 before inoculation to d10 after inoculation. Anti-Ly6G antibody was used to deplete neutrophils *in vivo* from -d8 before inoculation to d7 after inoculation, thus depleting neutrophils in the early stage of metastasis. The mice ($n = 6$ per group) were sacrificed on d21 after inoculation. Metastatic nodules on the surface of lungs were counted. (**D**) Early depletion of neutrophils favors the formation of micrometastases by T/H/H-B16F0 cells. Mice were inoculated with T/H/H-B16F0 cells (5×10^5/mouse). Anti-Ly6G antibody was used to deplete neutrophils *in vivo* from -d8 before inoculation to d7 after inoculation. The mice then received the i.v. injection of 14,15-EET (30 μg/kg), once every two days, form d22 to d40 after inoculation. The mice ($n = 6$ per group) were sacrificed on d42 after inoculation. Metastatic nodules on the surface of lungs were counted. (**E**) Increasing EET production *in vivo* promotes tumor cell metastasis. Mice were inoculated with B16F1 cells (5×10^5 cells/mouse) by injection via tail vein. The mice were treated with sEH inhibitor *t*-AUCB (oral gavage, 10 mg/kg/d), and/or 14,15-EEZE (i.v. injection, 30 μg/kg/2d), from -d10 before inoculation to d10 after inoculation. Anti-Ly6G antibody was used to deplete neutrophils *in vivo* from -d8 before inoculation to d7 after inoculation, thus depleting neutrophils in the early stage of metastasis. The mice ($n = 6$ per group) were sacrificed on d21 after inoculation. Metastatic nodules on the surface of lungs were counted. (**F**) Control mice (N) and the mice inoculated with T/H/H-B16F0 cells (T) were untreated or treated with 14,15-EET. On d42 after inoculation, neutrophils were isolated from the peritoneal cavity of mice after recruitment as described in Methods. The expression of *Stat3* gene was detected by real-time RT-PCR and Western blot. The relative level of STAT3 to β-actin was calculated after densitometric analysis of Western blots. (**G**) Mice were untreated or treated with 5,6-EET, 8,9-EET, 11,12-EET, and 14,15-EET (i.v. injection, 30 μg/kg) for 10 days, once every two days. The levels of serum G-CSF and IL-6 in naive mice and EET-treated mice were detected by ELISA. (**H**) 14,15-EET cooperates with G-CSF/IL-6 to modulate the expression of *Mmp9* and *Trail* genes in neutrophils. Neutrophils were isolated from bone marrow of naive mice, and stimulated with 14,15-EET (100 nM) and/or G-CSF/IL-6 (50 ng/ml of each) for 12 h. The expressions of *Mmp9* and *Trail* genes in neutrophils were detected at mRNA level by real-time RT-PCR. $*p < 0.05$, $**p < 0.01$.

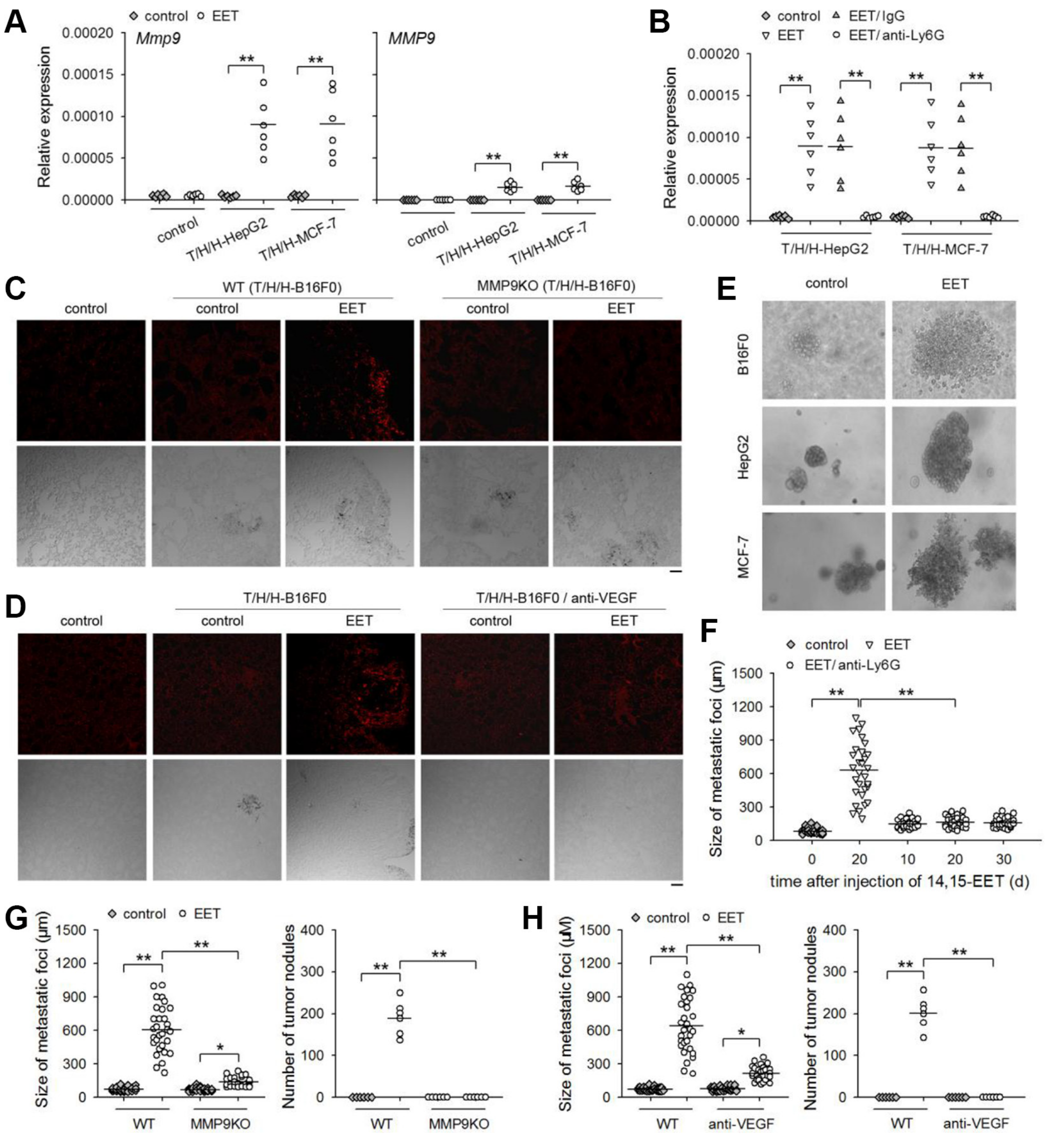

Supplementary Figure S8: The effect of MMP-9 on the development of dormant micrometastases. (**A**) Control mice and the mice inoculated with T/H/H-HepG2 cells or T/H/H-MCF-7 cells (n = 6 per group) were untreated or treated with 14,15-EET. The expression of mouse *Mmp9* gene and human *MMP9* gene in lung tissues was detected by real-time RT-PCR on d42 after inoculation. To compare the relative levels of human *MMP9* mRNA and mouse *Mmp9* mRNA in the lung tissues of mouse, the *MMP9* mRNA was quantified against mouse *Gapdh* mRNA. (**B**) Mice were inoculated with T/H/H-HepG2 or T/H/H-MCF-7, and treated with 14,15-EET. Anti-Ly6G antibody was used to deplete neutrophils *in vivo* when the mice were treated with 14,15-EET. The mice (n = 6 per group) were sacrificed on d42 after inoculation. The expression of mouse *Mmp9* gene in lung tissues was detected by real-time RT-PCR. (**C**) WT mice and MMP-9$^{-/-}$ mice (MMP9KO) were inoculated with T/H/H-B16F0 cells. The mice (n = 6 per group) were untreated or treated with 14,15-EET, and were sacrificed on d42 after inoculation. Lung tissue sections were prepared and stained for CD34 to identify microvessels. Representative photographs are shown. Bar, 50 µm. The WT mice without inoculation were also used as control. (**D**) Mice were inoculated with T/H/H-B16F0 cells. The mice (n = 6 per group) were untreated or treated with 14,15-EET. Anti-VEGF antibody (Genentech, San Francisco, CA) was used (i.p. injection, 125 µg/mouse, once every three days for six times) to block VEGF *in vivo* when the mice were treated with 14,15-EET. The mice were sacrificed on d42 after inoculation. Lung tissue sections were prepared and stained for CD34 to identify microvessels. Representative photographs are shown. Bar, 50 µm. The WT mice without inoculation were also used as control. (**E**) Tumor cells were untreated (control) or treated with 14,15-EET (100 nM) for 10 days. The cells were then cultured in soft agar for 3 weeks in absence (control) or presence of 14,15-EET. The representative colonies were photographed. (**F**) The mice inoculated with T/H/H-B16F0 cells were treated with 14,15-EET, starting from d21 after inoculation. Neutrophils were not depleted or depleted *in vivo* with anti-Ly6G antibody when the mice were treated with 14,15-EET. The mice (n = 6 per group) were sacrificed on the indicated time after 14,15-EET-treatment. Metastatic foci in tissue sections (H&E staining) were measured. (**G**) WT mice and MMP-9$^{-/-}$ mice (MMP9KO) were inoculated with T/H/H-B16F0 cells. The mice (n = 6 per group) were untreated or treated with 14,15-EET, and were sacrificed on d42 after inoculation. Lung tissue sections were prepared and subjected to H&E staining. Metastatic foci in tissue sections were measured (left). Metastatic nodules on the surface of lungs were also counted (right). (**H**) The mice (n = 6 per group) inoculated with T/H/H-B16F0 cells were untreated or treated with 14,15-EET. Anti-VEGF antibody was used to block VEGF *in vivo* when the mice were treated with 14,15-EET. The mice were sacrificed on d42 after inoculation. Metastatic foci in tissue sections were measured (left). Metastatic nodules on the surface of lungs were counted (right). *p < 0.05, **p < 0.01.

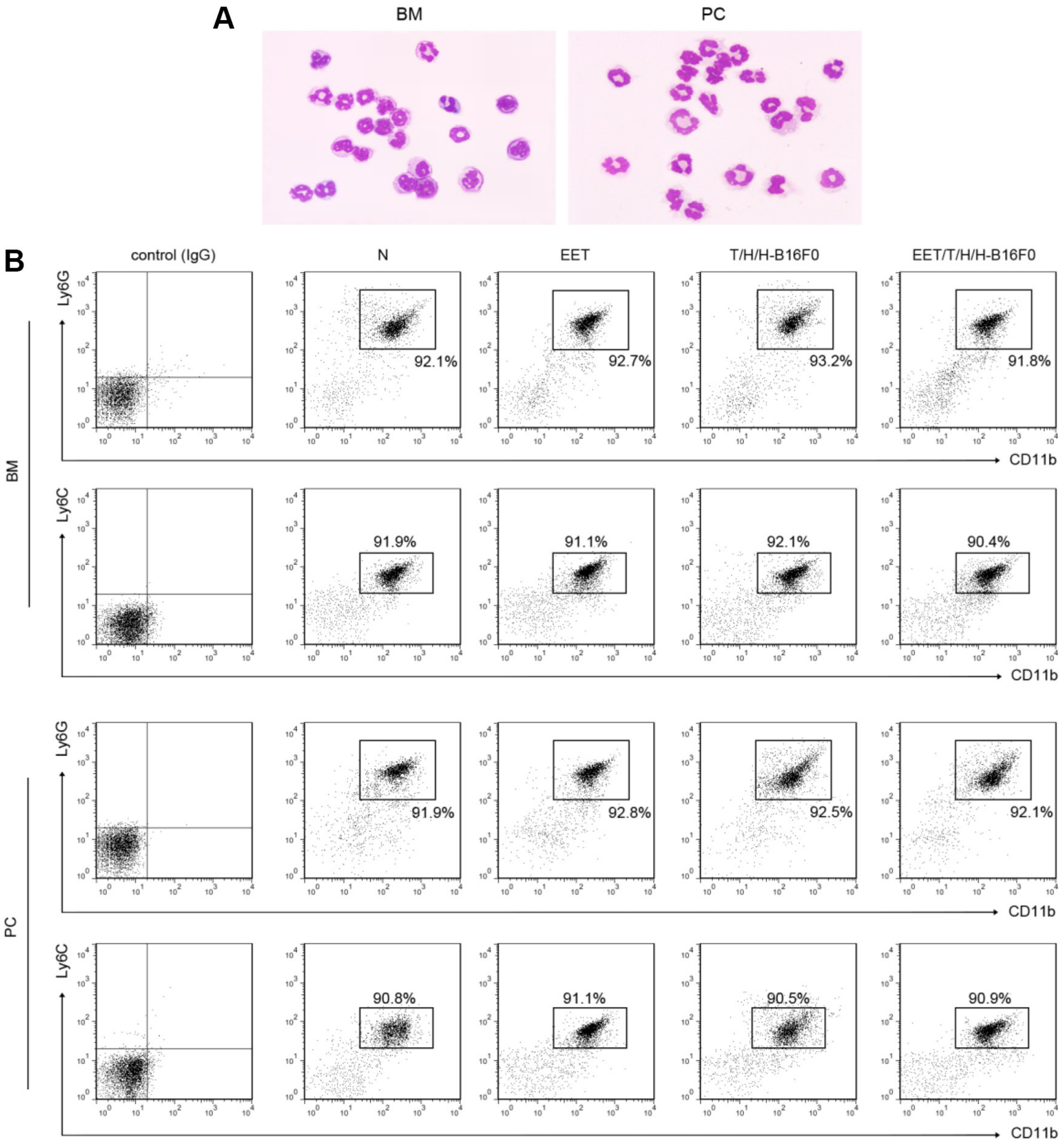

Supplementary Figure S9: Analysis of the isolated neutrophils. (**A**) Neutrophils were isolated from bone marrow (BM) and peritoneal cavity (PC) of mice. The cells were identified by Giemsa-Wright stain. (**B**) Neutrophils were isolated from bone marrow and peritoneal cavity of the indicated mice. The cells were stained with PE-Cy7-anti-mouse CD11b and PE-anti-mouse Ly6G or PE-anti-mouse Ly6C antibodies, and used for flow cytometric analysis.
